# Imaging of thoracic hernias: types and complications

**DOI:** 10.1007/s13244-018-0670-x

**Published:** 2018-11-27

**Authors:** Abhishek Chaturvedi, Prabhakar Rajiah, Alexender Croake, Sachin Saboo, Apeksha Chaturvedi

**Affiliations:** 1 Imaging Science, University of Rochester Medical Center, 601, Elmwood Avenue, Rochester, NY 14642 USA; 2 Radiology, University of Texas Southwestern Medical Center, Dallas, TX USA

**Keywords:** Lung hernia, Diaphragmatic hernia, Sternal dehiscence, Pericardial hernia

## Abstract

**Abstract:**

Thoracic hernias are characterised by either protrusion of the thoracic contents outside their normal anatomical confines or extension of the abdominal contents within the thorax. Thoracic hernias can be either congenital or acquired in aetiology. They can occur at the level of the thoracic inlet, chest wall or diaphragm. Thoracic hernias can be symptomatic or fortuitously discovered on imaging obtained for other indications. Complications of thoracic hernias include incarceration, trauma and strangulation with necrosis. Multiple imaging modalities are available to evaluate thoracic hernias. Radiographs usually offer the first clue to the diagnosis. Upper gastrointestinal radiography can identify bowel herniation and associated complications. CT and occasionally MR can be useful for further evaluation of these abnormalities, accurately identifying the type of hernia, its contents, associated complications, and provide a roadmap for surgical planning. In this article, we review the different types of thoracic hernias and the role of imaging in the evaluation of these hernias.

**Teaching Points:**

*• Protrusion of lung contents beyond the anatomic confines of the thorax constitutes a hernia.*

*• Complications of thoracic hernias include incarceration, trauma and strangulation with necrosis.*

*• Multiple imaging modalities are available to evaluate thoracic hernias.*

*• CT is the imaging modality of choice for identifying thoracic hernias and their complications.*

*• Imaging can provide a roadmap for surgical planning.*

**Electronic supplementary material:**

The online version of this article (10.1007/s13244-018-0670-x) contains supplementary material, which is available to authorized users.

## Introduction

**Fig. 1:**
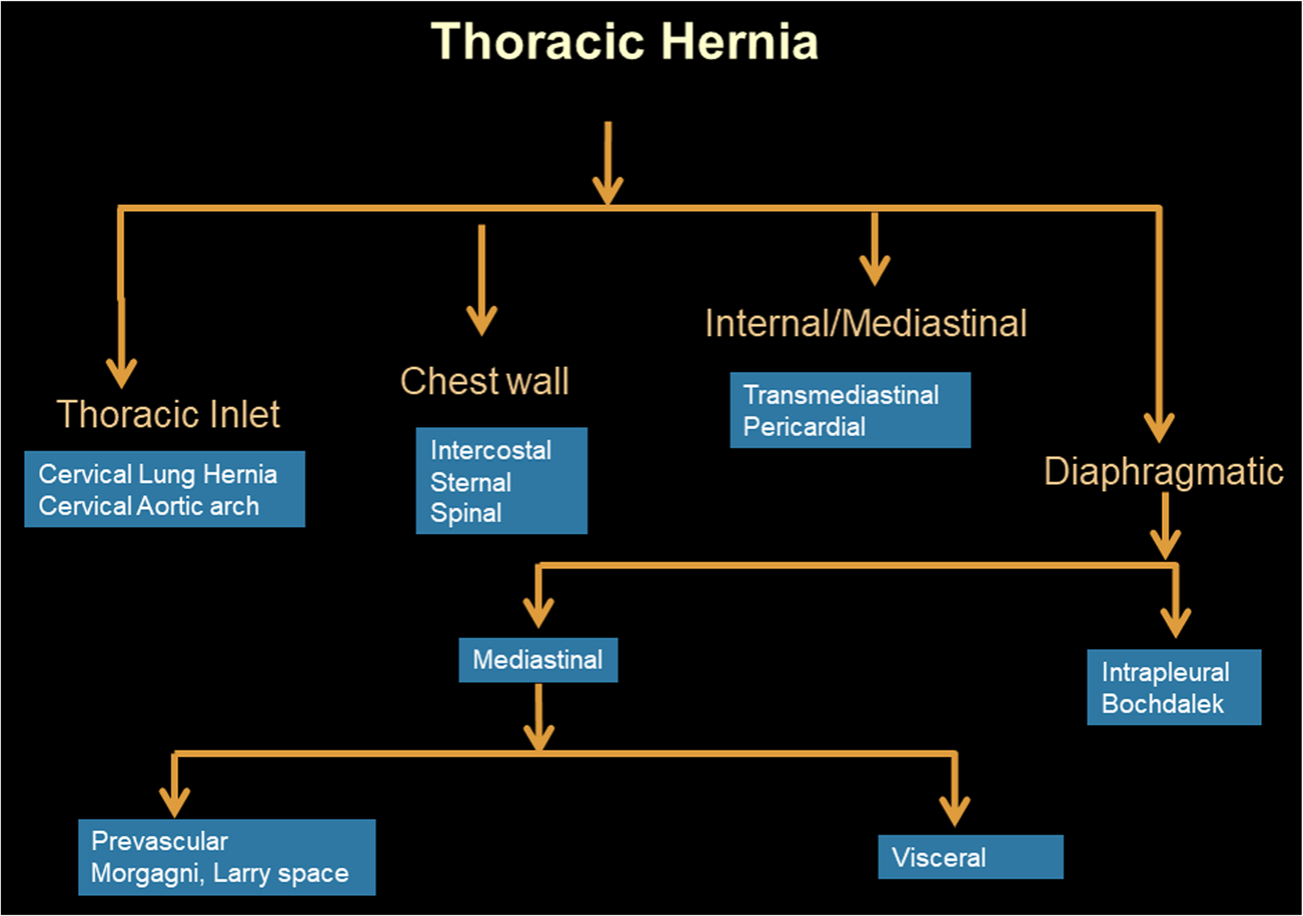
Flowchart depicting the different types of thoracic hernias seen on imaging

Thoracic hernias are characterised by either protrusion of the thoracic contents outside their normal anatomical confines or extension of the abdominal contents into the thorax. Thoracic hernias can be congenital or acquired in aetiology. When acquired, these are usually post-traumatic or post-surgical (Fig. 1). They can occur at the level of the thoracic inlet, chest wall or diaphragm [1]. Diaphragmetic hernias can be either mediastinal or intrapleural. These hernias can be symptomatic or incidentally detected during routine imaging of the chest or abdomen for other indications. Complications of thoracic hernias include incarceration, trauma and strangulation with necrosis. These complications can mimic cardiovascular or gastrointestinal causes of chest and abdominal pain, some of which often necessitate urgent intervention or surgery.

## Imaging thoracic hernias

Different imaging modalities can be useful in identification of thoracic hernias, with their advantages and disadvantages (Table 1), including radiographs, upper gastrointestinal series, ultrasound, computed tomography (CT) and magnetic resonance imaging (MRI). The roles of imaging in the evaluation of hernias (Table 2) include establishing the diagnosis, characterising the type, delineating the extent, identifying the contents, detecting complications and providing a roadmap for intervention/surgery.

**Table 1:** Different imaging modalities used in the evaluation of thoracic hernias

	Advantages	Disadvantages	Indications
Chest radiograph	Inexpensive; readily available	Limited sensitivity and specificity	Reasonable first test screening
Ultrasound	Portable; inexpensive; widely available; real-time data	Operator dependent; limited by acoustic window in the chest, especially in large patients	Prenatal and paediatric group, where radiation dose is a concern
CT	Good spatial resolution providing anatomical detail; wide field of view; multi-planar reconstruction	Radiation exposure; potentially nephrotoxic iodinated contrast	Comprehensive evaluation of thoracic hernia-establishing diagnosis; characterising the type; identifying contents; detecting complications; providing road map to intervention/surgery
MRI	Good spatial resolution; excellent contrast resolution; multi-planar acquisition; wide field of view	Limited to a few of centres; time-consuming; expensive; contraindications	Further classification of diaphragmatic hernias and hernias, particularly those involving cardiac structures
Fluoroscopy	Real-time anatomical and physiological information	Radiation exposure; requires patient cooperation	Evaluation and classification of hiatal hernias; gastrointestinal leaks

**Table 2:** Summary of different types of thoracic hernias, their imaging findings, mimics, and brief description of the treatment

Type	Imaging features	Mimics	Treatment
Superior thoracic aperture			
Apical/cervical lung	CXR: lateral deviation of trachea by unilateral lucency CT/MRI: supraclavicular protrusion of lung posterior to the subclavian vessels. Enlargement with Valsalva manoeuvre	Supraclavicular emphysema, apical bulla	Imaging follow-up in asymptomatic patients Congenital hernias in infants may resolve spontaneously Elective surgical repair in symptomatic patients or those with incarcerated hernia
Cervical aortic arch	CXR: absence of aortic knob, tracheal deviation to contralateral side CT/MRI: elongated aortic arch extending into the neck	Aneurysm of carotid arteries Vascular rings	Increased risk of dilation and aneurysm that may require follow-up imaging, endovascular or surgical repair
Chest wall			
Intercostal	CT: protrusion of lung parenchyma or other viscera beyond the intercostal space	Chest wall emphysema Eloesser reconstruction	No intervention for asymptomatic hernia Elective surgical repair for incarcerated hernia Emergent surgical repair for strangulated hernia
Sternal	CXR: lateral view may identify presternal opacity CT: protrusion of pericardium, cardiac chambers or great vessels through the sternal dehiscence	Postoperative sternal infection, haematomas or seroma Pericardiocutaneous fistula	Elective surgical repair with musculocutaneous grafts. Radical sternectomy for post-sternotomy mediastinitis
Spinal	CXR: widening of mediastinum, paraspinal opacity, associated vertebral anomalies CT/MRI: protrusion of meninges with CSF and occasionally spinal cord or nerves into posterior mediastinal, pleura or chest wall	Foregut duplication Cyst Cystic neoplasms	Elective surgical repair
Transmediastinal			
Transmediastinal	CXR/CT: lung herniation across anterior-posterior junction lines Pleural sac or fluid herniation across posterior junction line	Post-pneumonectomy Atelectasis from bronchial obstruction	May require placement of tissue expander for bronchial narrowing/stenosis
Transdiaphragmatic			
Intrapleural	CXR: bowel loops in the hemithorax, elevated hemidiaphragm, NG tube above the left hemidiaphragm CT: direct sign: Defect in the diaphragm, dangling diaphragm Indirect: herniation of abdominal fat or viscera into the pleural cavity, collar sign	Diaphragmatic mass, lipomas	Laparotomy with repair during the acute phase Transthoracic or thoracoabdominal approach for chronic hernia
Mediastinal	CXT: opacity at the anterior cardiophrenic angle. CT/MRI: small defect in between pars sternalis and pars costalis with herniation of omentum or bowel loops	Pericardial cyst, prominent pericardial fat or mediastinal lipomatosis	No treatment for asymptomatic hernia Elective repair for herniated viscera or bowel
Pericardial	CXR: air fluid level from herniated bowel in the retrosternal region CT/MRI: herniation of abdominal organs, omentum or bowel loops into the pericardium	Pericardial haematoma, primary tumour, metastasis	Elective repair for herniated viscera or bowel
Type I hiatal hernia	Oesophagogram/ CT: displacement of oesophagogastric junction into thorax		No surgery for asymptomatic hernia Medical treatment of reflux disease Antireflux procedure
Type II hiatal hernia	Oesophagogram/ CT: GE junction in normal position, fundus herniates into thorax	Epiphrenic or traction diverticulum Oesophageal fistula	Symptomatic hernia: elective surgical repair
Type III hiatal hernia	Both GE junction and fundus herniate	Epiphrenic or traction diverticulum Gastric or oesophageal fistula	Elective surgical repair
Type IV hiatal hernia	Other viscera also herniate in addition to stomach	Postoperative appearance after upper gastrointestinal surgery	Elective surgical repair
Sub-diaphragmatic			
Sub-diaphragmatic	Extension of the abdominal wall hernia through the superficial and deep fascia into thorax	Surgically created vascular or bowel conduits	Elective surgical repair

*CXR* chest radiograph, *GE junction* gastro-oesophageal junction

### Radiographs

Chest radiographs (CXR) are usually the first imaging modality used in adults with any suspected thoracic pathology and may offer the first clue to a thoracic hernia.

### Gastrointestinal tract radiography

Gastrointestinal contrast radiography under fluoroscopy is a useful modality in identifying bowel herniation into the thorax. Upper gastrointestinal imaging with oral contrast (UGI) is useful in identification and classification of the hiatal hernias. Lower gastrointestinal radiography (contrast enema) is useful in identifying herniation of the large bowel into the thorax and complications of large bowel conduits. In patients with suspected bowel perforation, water-soluble contrast is initially used for both UGI and enema examinations. At our institution, low osmolar non-ionic contrast such as iohexol 350 mgI/ml (Omnipaque 350, GE Healthcare, Princeton, NJ, USA) in adults and Optiray 240 (Ioversol 240 mgI/ml, Guerbet LLC, Bloomington, IN, USA) in children < 17 years is used. We limit the use of oral barium to assessing any leaks in patients with large body habitus or overlying metal hardware when images with water-soluble or low-osmolar agents are suboptimal. Some radiologists prefer not to use barium sulfate in young or debilitated older patients who may be at risk for aspiration or may need additional imaging [1, 2].

### Ultrasound

Ultrasound (US) is the modality of choice in the prenatal evaluation of congenital malformations and can identify congenital diaphragmatic hernias. It can be used in the paediatric age group, where the radiation dose is a concern. It is portable, inexpensive and widely available and provides real-time information. US can be a useful modality to confirm a superficial thoracic hernia.

### Computed tomography

CT is the cross-sectional imaging modality of choice in the identification and characterisation of thoracic hernias in adults. CT accurately identifies and classifies thoracic hernias as well as the contents of the hernia sac. On CT, the dimensions of the hernia sac and size of the defect can be measured. CT is also useful for assessment of any associated complications. The modern multidetector CT (≥ 16 detector rows) yields isotropic voxels that can be displayed in multiple different planes. Image analysis of these axial CT images can generate three-dimensional, volume- or surface-rendered images as well as minimum- and maximum-intensity projections for a more comprehensive review of the hernias. Most thoracic hernias can be identified and classified on a single venous phase contrast-enhanced CT of the thorax. In our practice, we use oral contrast in those patients who have suspected bowel herniation or who are also undergoing a concurrent abdomen CT.

### Magnetic resonance imaging

MRI can identify non-acute pericardial and diaphragmatic hernias. MRI without intravenous contrast can characterise the hernia in patients who cannot receive contrast for CT (renal failure, history of anaphylaxis after previous contrast medium administration, etc.). Multiple acquisitions can be obtained, which can help characterise herniation of the abdominal contents such as the liver or spleen since these can be suboptimally characterised on a single-phase contrast-enhanced CT examination [3]. Steady-state free-precession sequences are used to identify in utero foetal congenital diaphragmatic hernias. T2-weighted axial, coronal and sagittal images are helpful in identifying defects in the diaphragm or the chest wall. Contrast-enhanced images after injection of intravenous gadolinium can characterise contents of the hernia sac and also assess for any complications in the herniated solid viscera. Time-resolved dynamic imaging during inspiration, expiration and the Valsalva manoeuvre can be useful to show changes in the hernia with different abdominal or intrathoracic pressures.

## Thoracic hernias

### Hernias at the superior thoracic aperture

The superior thoracic aperture is formed by the manubrium, both the first ribs and the first thoracic vertebra. The lung apices are covered by the apical parietal pleura and Sibson fascia, which extends from C7 to the first ribs. Apices of the lungs extend only for about 2.5–5 cm superior to the superior border of the sternal end of the first rib. Any extension of the thoracic contents above the thoracic inlet constitutes cervical or apical hernia.

#### Cervical lung herniation

**Fig. 2:**
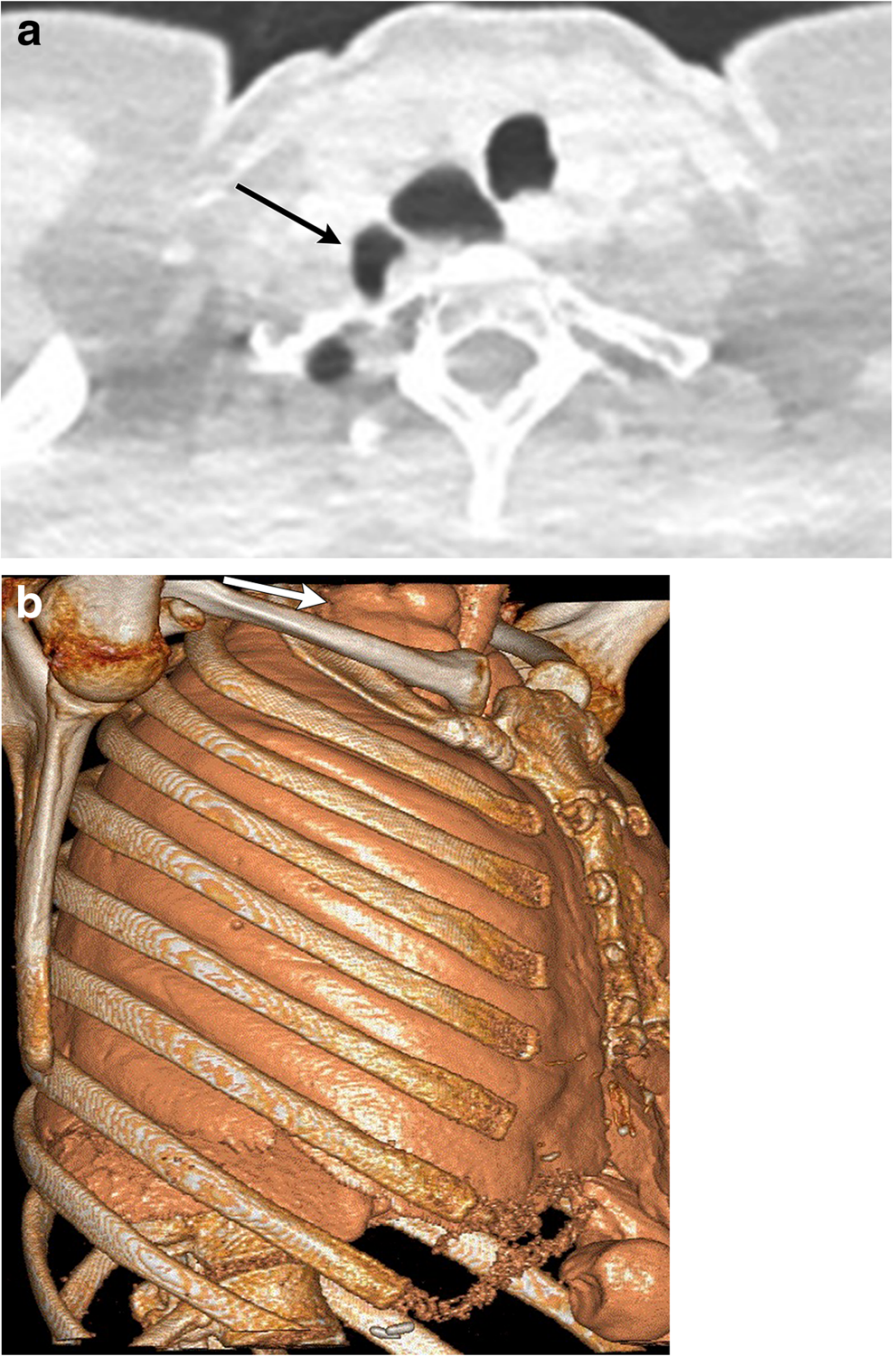
**a, b** A 67-year-old female being evaluated for pulmonary nodules and recent acute exacerbation of reactive airway disease. Axial CT (**a**) demonstrates the apicoposterior segment of right upper lobe (black arrow) extending into the cervical region. Volume-rendered image (**b**) clearly depicts the herniated lung above the clavicle and first rib (white arrow). Herniated lung causes a smooth impression on the trachea. These can cause tracheal deviation or compression

Herniation of the lungs in the cervical region is rare and mostly described in case reports. Lung hernias can be cervical, thoracic or diaphragmatic. Cervical hernias can be seen in children with asthma [5], in adults with obstructive lung disease and after surgery [4] (Fig. 2a, b) or trauma. Post-traumatic hernia can form as a sequela of tears in the Sibson fascia with a well-defined hernia sac. Hernia can be due to chronically elevated intrathoracic pressure present as laxity of the suprapleural membrane with no hernia sac [5]. These can be unilateral or bilateral.

On radiographs, unilateral cervical lung hernia is seen as a unilateral lucency at the level of the thoracic inlet with contralateral tracheal deviation [8]. Postoperative subcutaneous emphysema can mimic a cervical lung hernia but can be differentiated on CT. Asymptomatic supraclavicular lung hernias do not require surgical repair [6] and most paediatric hernias resolve spontaneously. Surgical repair of the hernias may be necessary when complications are present, such as neurological pain from neural compression [5].

#### Cervical aortic arch

**Fig. 3:**
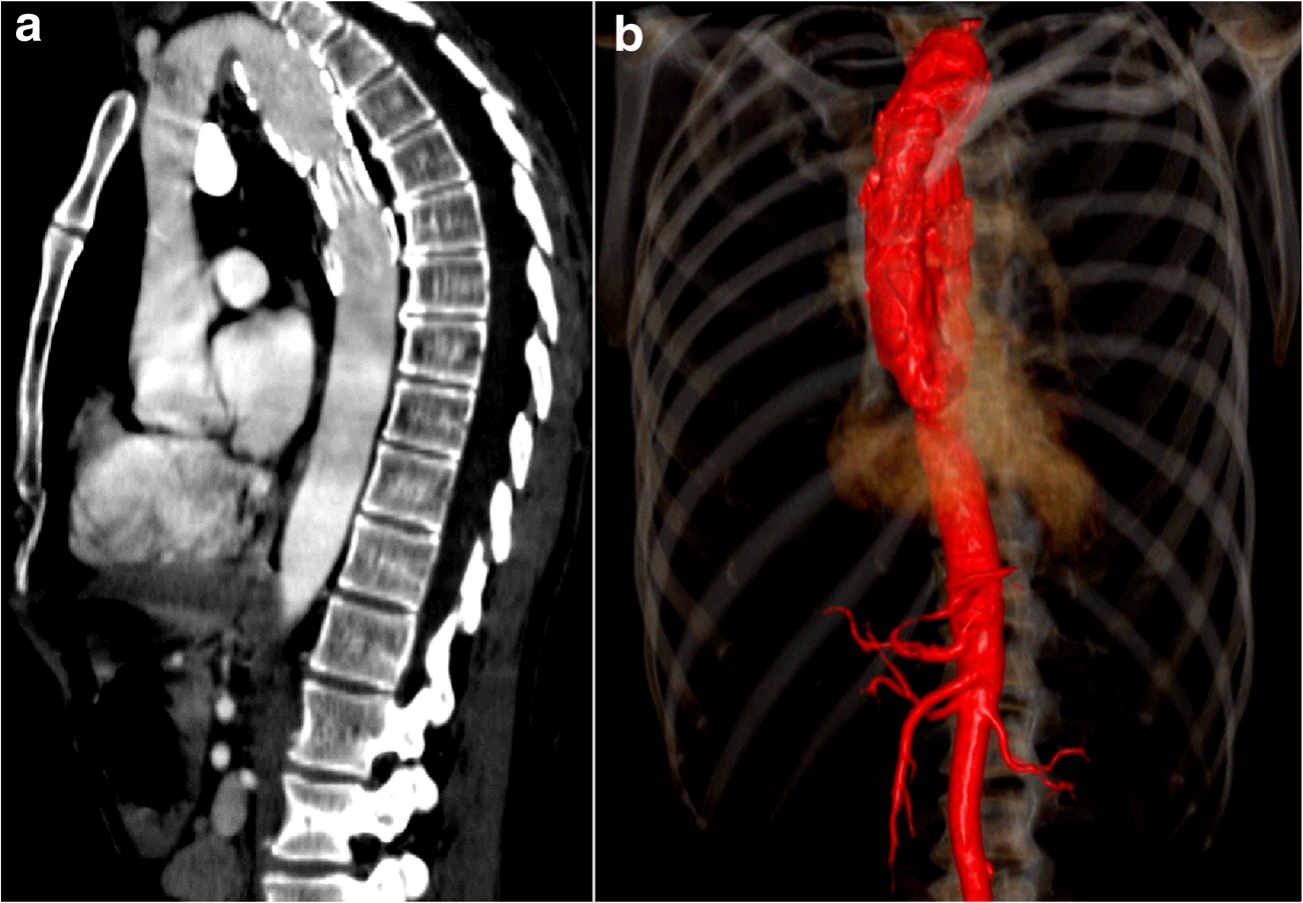
**a, b** A 69-year-old female with a remote history of treated small-cell carcinoma and stent graft placement of the descending aorta presenting with slowly progressive left supraclavicular mass. Axial contrast-enhanced CT (CECT) image (**a**) above the level of the sternoclavicular joint and volume-rendered image (**b**) identifies a high, dilated and elongated aortic arch extending into the left cervical region. These aortic arches can dilate with aneurysm formation, which can cause a mass effect on the surrounding structures in the thoracic inlet

Cervical aortic arch is a rare congenital anomaly in which the aortic arch extends into the soft tissues of the neck (Fig. 3a, b). The aortic arch normally develops from the right fourth branchial arch. However, in cervical aortic arch, it develops from the third arch, with the fourth arch being atretic [7]. Cervical aortic arch is more common on the left [8]. Cervical aortic arch can present as a pulsatile supraclavicular mass. On CT and MRI, the arch extends above the sternum into the cervical region. This can dilate and form an aneurysm. Dilation can be due to abnormal development, abnormal connective tissue or altered haemodynamics with high aortic wall stress and trauma [9]. Cervical aortic arch aneurysms are treated with endovascular repair or using an interposition graft after sternotomy.

### Chest wall hernia

The chest wall is comprised of skin, superficial fascia, deep fascia, muscles and the thoracic skeleton (ribs, sternum, clavicle, scapula and vertebral bodies). The intercostal space has three intercostal muscles (external, internal and innermost), which are thin or deficient, anteriorly near the sternum and posteriorly near the vertebral bodies. There is an extra pleural space, which lies between the inner surface of the ribs and the parietal pleura [10]. The chest wall hernias can be intercostal, sternal or spinal in location.

#### Intercostal lung hernia

Intercostal lung hernia is a protrusion of the lung parenchyma through a defect in the chest wall. Only a few cases have been reported in the literature on these hernias [11]. Intercostal lung hernia can be congenital (associated with costal cartilage defect or rib hypoplasia), spontaneous (sudden increase in intrathoracic pressure such as during coughing, sneezing, blowing a musical instruments, etc.) or, more commonly, secondary to trauma or inadequate healing after thoracic surgery (thoracotomy, thoracoscopy, minimally invasive cardiac surgery) [11–13]. Lung is the most common content of an intercostal hernia sac. Intercostal hernia can be asymptomatic or present with localised chest pain in patients with prior thoracotomy or chest tube placement.

**Fig. 4:**
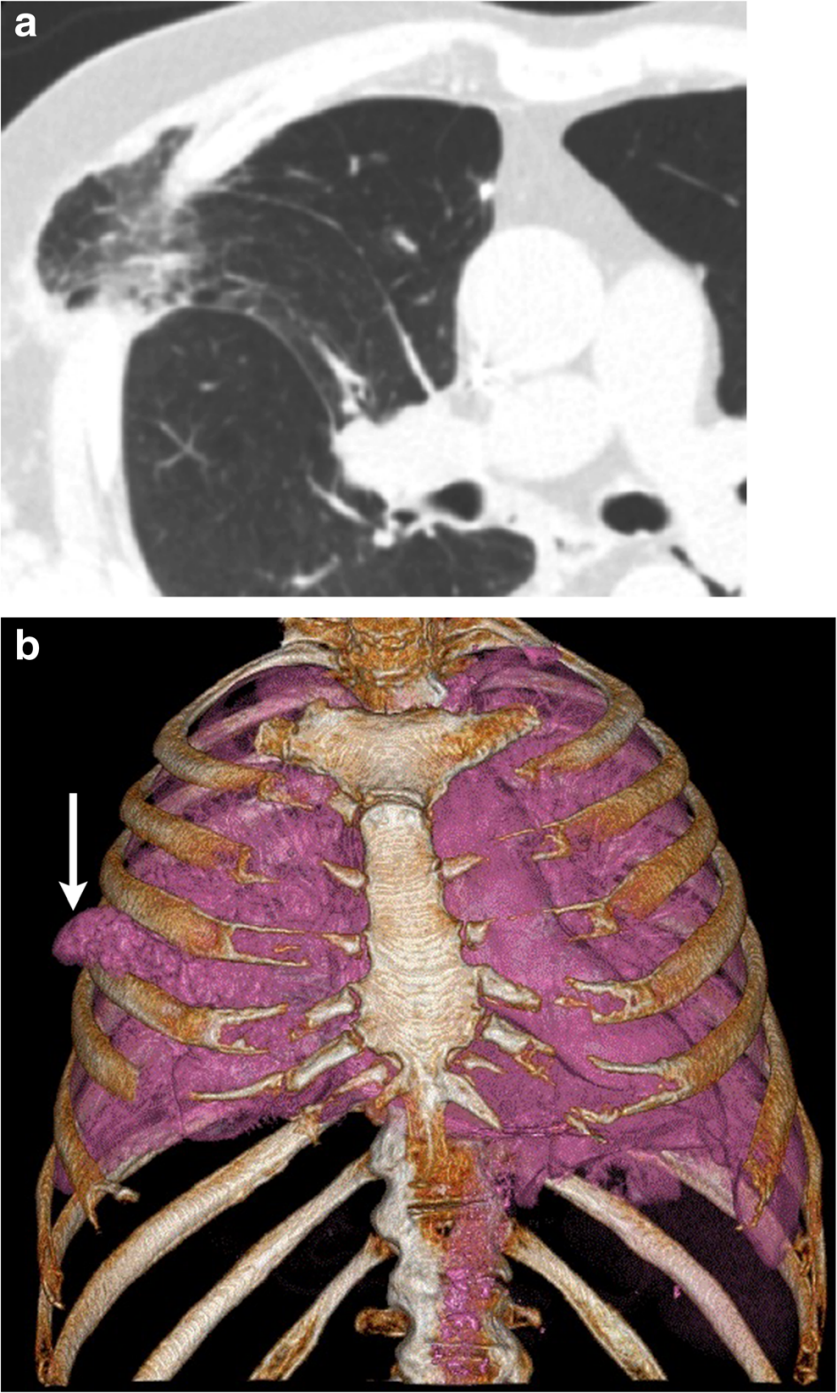
**a, b** A 70-year-old female with a history of intercostal chest tube drainage for pleural effusion. Axial CT (**a**) and volume-rendered image (**b**) in a patient with previous right upper lobe wedge resection for a stage I lung cancer identifies focal intercostal herniation of the right upper lobe (white arrow). The neck of the hernia is narrow, indicating incarceration. In addition, there is abnormal orientation of the bronchovascular pedicle with narrowing of the bronchiole. Ground-glass opacities in the herniated lung indicate atelectasis (Movie 1). Elective repair is recommended for these hernias unless asymptomatic

Intercostal hernias can be missed on plain radiographs [11]. On CT and MRI, protrusion of lung along with pleura is identified through a defect in the chest wall, with only a thin layer of fascia and skin covering the herniated lung. Atelectasis or scar can be present in the herniated lung. Maximum intensity projection (MIP) images can be useful to evaluate for vascular compression, minimum intensity projections (MinIP) images can be useful to evaluate for airway compression and volume rendering can be useful in presurgical planning (Fig. 4a, b). Bioprosthetic implants are preferred over rigid medical implants for closure of such hernias [14]. Hernias presenting with pain or lung entrapment require reconstruction with a surgical mesh graft to close the defect as the entrapped lung can undergo strangulation and recurrent infections [12].

#### Intercostal hernia of the abdomen viscera

**Fig. 5:**
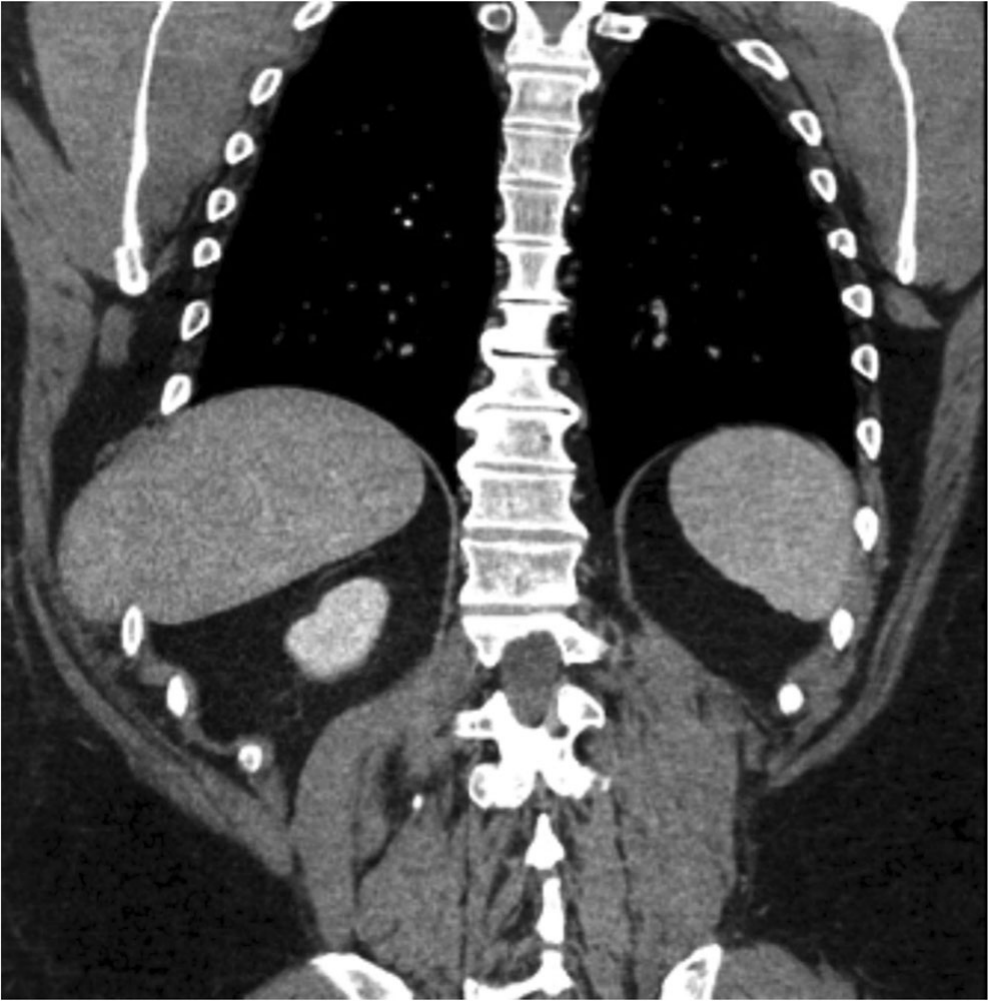
Coronal CT in a patient with a remote history of trauma demonstrates intercostal herniation of the liver

**Fig. 6:**
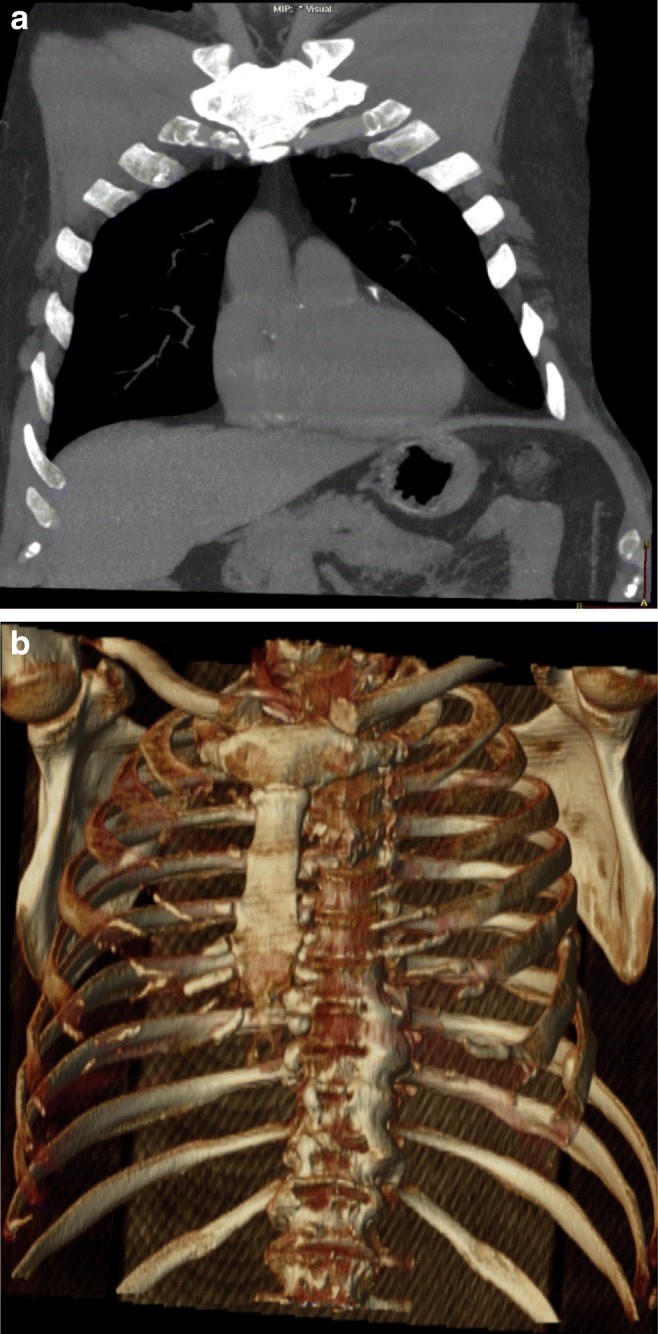
**a, b** Coronal MIP demonstrates herniation of the mesenteric fat through the left 7th–8th intercostal space (**a**) with volume-rendered reconstruction (**b**)

Abdominal intercostal hernia is a rare acquired hernia occurring through defects in the diaphragm and adjacent intercostal muscles [15]. It is usually secondary to penetrating or blunt thoracoabdominal injuries and can be seen in patients with COPD, osteoporosis and muscle weakness. These can occur even after minor events such as coughing or heavy lifting. Clinically, these hernias can be easily identified because of their superficial soft tissue location but these can be missed on CXR. The liver can herniate on the right (Fig. 5) and spleen or peritoneum (Fig. 6a, b) on the left side.

#### Sternal dehiscence and hernia

Sternal dehiscence is a rare but grave complication of cardiac surgery. It represents separation of the bony sternum and can occur in 0.2–5% of patients after median sternotomy. It can be due to primary non-union, poor wound healing or premature overexertion [16] and can be associated with infections and mediastinitis. Early dehiscence is difficult to identify clinically. Cardiovascular structures commonly herniate through the dehiscence. Gastric herniation has also been reported [22], particularly a median sternotomy that extends into the epigastric region and weakens the upper anterior abdominal wall [23].

**Fig. 7:**
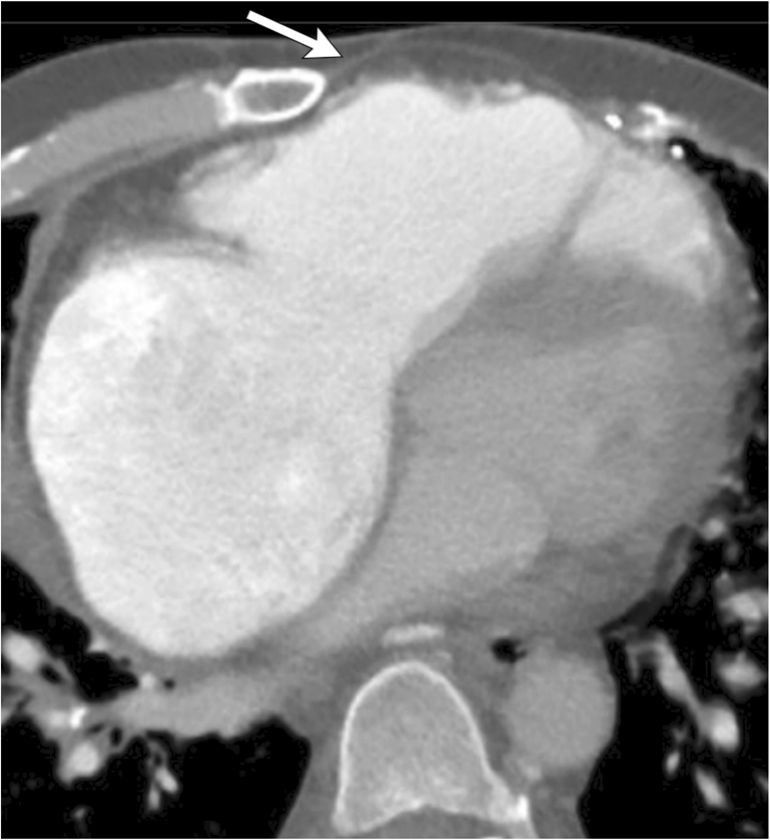
A 55-year-old male with a history of ascending aortic aneurysm status post-ascending aortic graft replacement. Axial CECT demonstrates sternal dehiscence with herniation of the right ventricle through the sternal defect. Only a thin layer of pericardium (white arrow) separates the skin from the myocardium

On CXR, altered configuration of sternal wires can be suggestive of impending dehiscence [17]. En bloc displacement of the wires indicates gross separation of sternal margins. A midsternal stripe thicker than 3 mm on CXR is also suggestive of sternal dehiscence [18]. The aorta, pulmonary artery, right ventricle or right ventricular outflow tract can herniate through the sternal dehiscence (Fig. 7) and can be identified on CT or MRI. A thin layer of pericardium, subcutaneous fat and skin overlies the myocardium, providing inadequate coverage with risk of myocardial injury with even minor trauma. Sternal debridement with flap coverage constitutes the mainstay of therapy [19].

The immunological and angiogenic properties of a greater omental graft render it useful for treatment of mediastinitis and wound infection. Mediastinal placement of the greater omentum represents an acquired hernia as the omentum is harvested from the abdomen and repositioned into the thorax through a surgically created transdiaphragmatic opening. Omental grafts are sometimes also used to buttress a post-pneumonectomy bronchial stump and to fill in the post-pneumonectomy space.

#### Pericardial hernia

**Fig. 8:**
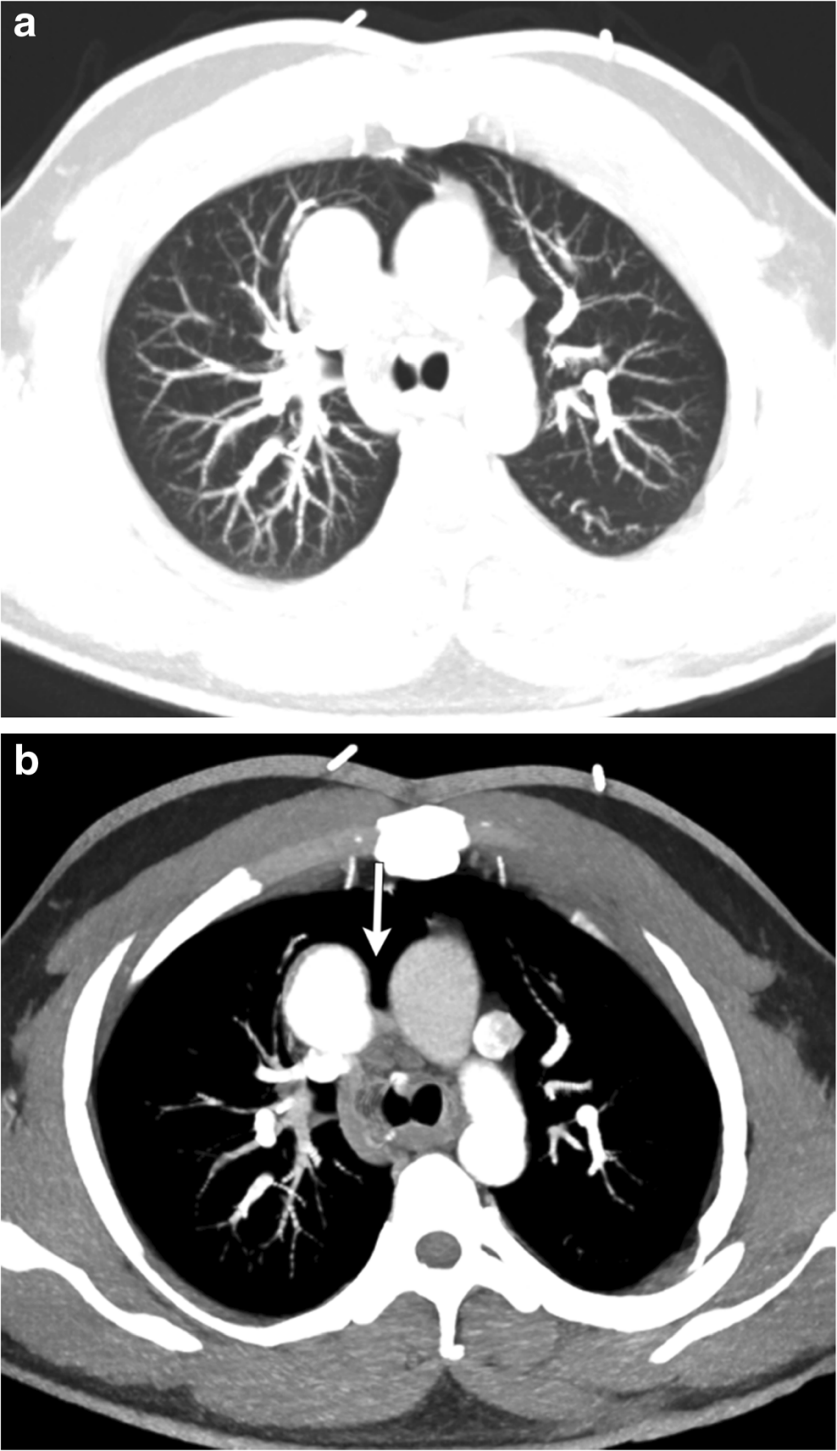
**a, b** A 26-year-old male with a history of Shone’s complex, status post-coarctation repair, subaortic membrane resection and supravalvular mitral ring resection. Maximum intensity projection axial CECT images in the lung (**a**) and mediastinal (**b**) windows demonstrate focal herniation of the anterior segment of the right upper lobe (white arrow) between the ascending aorta and main pulmonary artery. This lung herniation is a result of focal absence of the pericardium

**Fig. 9:**
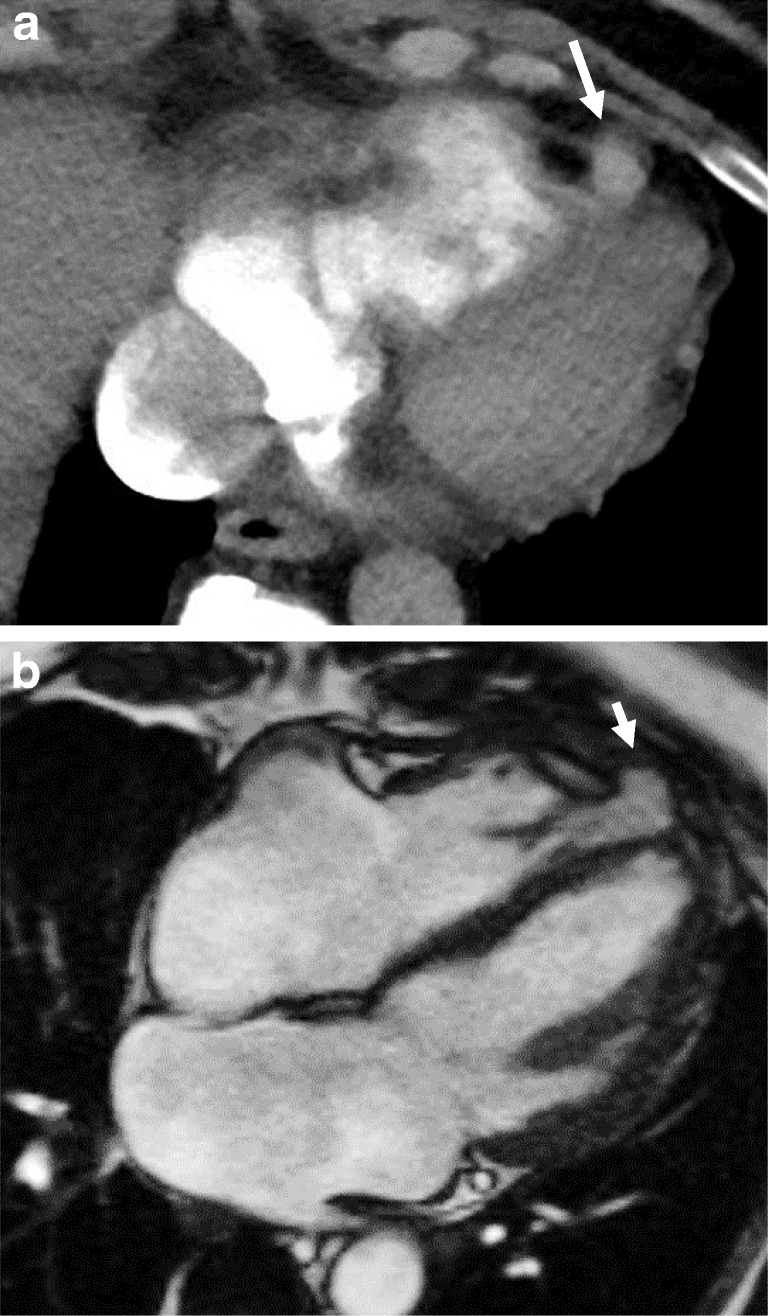
**a, b** CT (**a**) and MRI (**b**) images through the heart demonstrate focal hernia of the right ventricular myocardium through a congenital anterior pericardial defect (white arrow)

Pericardial defects can be congenital or acquired after pericardiectomy, lung or cardiac transplant or trauma. It can be either complete or partial. The incidence of congenital pericardial defects is reported to be < 1 per 10,000 based on autopsies [20]. Congenital absence of the pericardium results from abnormal early regression of the common cardinal vein, which leads to incomplete formation of the pleuropericardial membrane. Pericardial tears in blunt trauma most commonly occur along the left pleuropericardium parallel to the location of the phrenic nerve [21]. The true incidence of pericardial defects is likely underreported as many cases may remain asymptomatic [22]. Although congenital complete left-side defects are more common, partial defects tend to be symptomatic and have a higher incidence of complications. With a partial defect there can be herniation of the lung parenchyma into the pericardial defect. On imaging lung herniation can be identified (Fig. 8a, b) between the ascending thoracic aorta and main pulmonary artery [23]. Focal herniation of cardiac cambers (Fig. 9a, b) can also occur through such partial defects. The most common cardiac chamber to herniate is the left atrial appendage. The herniation is more prominent during systole.

#### Spinal hernia: thoracic meningocele

**Fig. 10:**
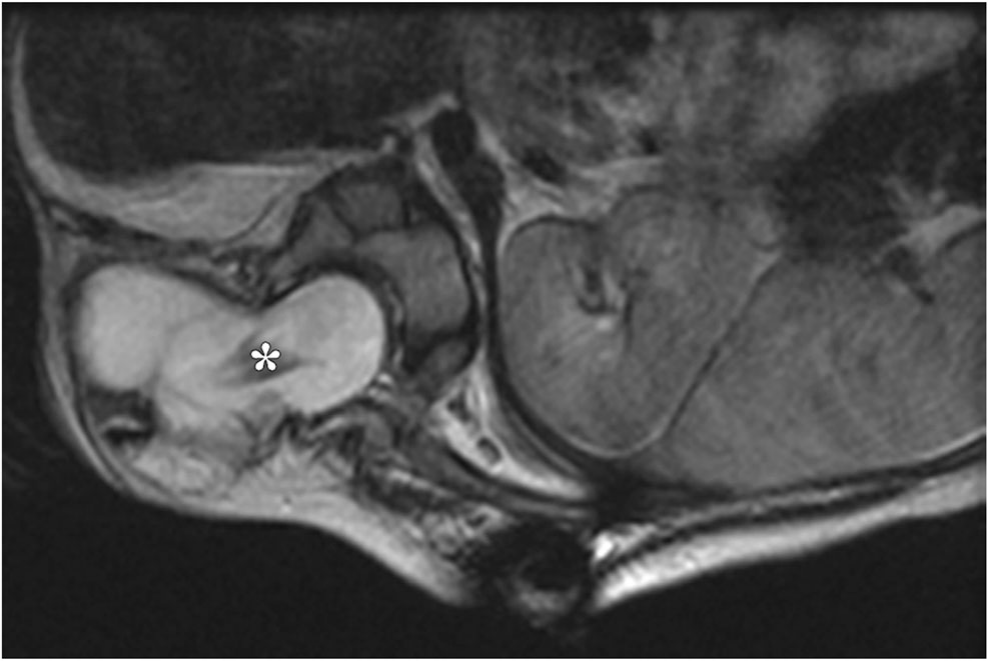
Axial T2-weighted MRI in a 1-month-old infant with a right posterior paravertebral mass. Non-contrast axial T2-weighted MRI identifies a right lateral myelomeningocele. Besides the herniated meninges, a neural placode (white star), which was not seen on CT (not shown), is also seen on the MRI, thus confirming this to be a lateral thoracic myelomeningocele

The contents of the spinal canal can herniate into the chest wall, pleura or posterior mediastinum, forming an intrathoracic meningocele [24]. Congenital meningocele is seen in patients with neurofibromatosis, while acquired meningocele occurs after laminectomy or associated with musculoskeletal deformities [25]. These are often asymptomatic. CXR can identify a soft tissue lesion in the posterior mediastinum. On CT and MRI, the herniation of meninges and CSF through an intervertebral foramen with cyst formation can be identified [28]. MRI is superior to CT in identification of the neural placode, which is present in myelomeningoceles but not in meningoceles (Fig. 10).

### Transmediastinal hernia

Transmediastinal hernia refers to the herniation of the pleural sac and its contents across the mediastinum to the opposite side. This entity is distinct from mediastinal displacement in which the entire mediastinum shifts towards one hemithorax. Herniation of the lung can be seen in patients with sequestration, scimitar syndrome or post pneumonectomy and commonly occurs across the anterior mediastinum, while herniation of the pleural sac and fluid commonly occurs across the posterior inferior mediastinum [26]. Trans-mediastinal herniation of the giant bulla has also been described [27].

**Fig. 11:**
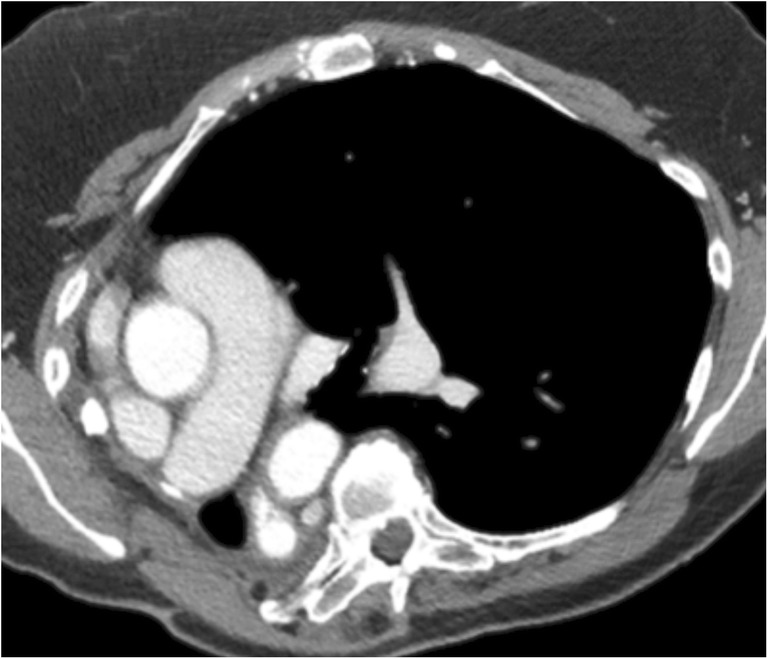
A 55-year-old female with adenocarcinoma of the right upper lobe extending to hila status post right pneumonectomy. Follow-up surveillance CECT demonstrates a mediastinal shift with left lung herniation into the right hemithorax

**Fig. 12:**
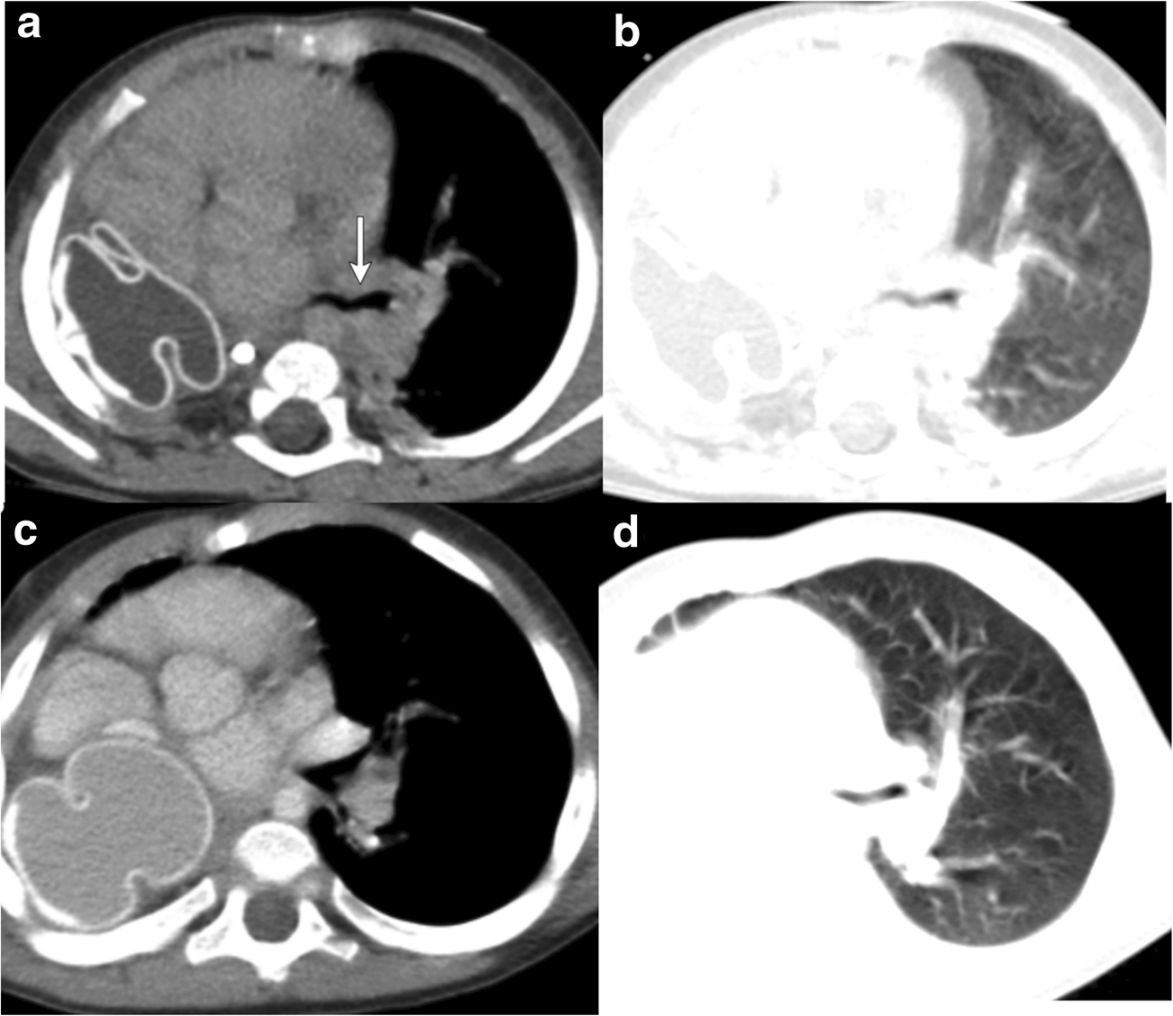
**a–d** A 5-year-old child with a history of right pneumonectomy for hypoplastic right lung and scimitar syndrome at the age of 1 year and now presenting with gradually increasing dyspnea. Axial CECT demonstrates a tissue expander in the right hemiothorax that was placed at the time of initial surgery (**a, b**). In addition, narrowing of the left bronchus is also seen, likely the cause of the post-pneumonectomy syndrome (white arrow). Follow-up axial CECT (**c, d**) with the upsized tissue expander in the right hemithorax demonstrates decreased narrowing of the left bronchus. In a growing child, these tissue expanders may need to be upsized over time to prevent these symptoms

On CXR, the anterior junction line is displaced. On the lateral radiograph, the hernia is seen as a retrosternal lucency that can mimic an anterior pneumothorax. CT is the modality of choice and identifies displacement of either the anterior or posterior junction line without displacement of the mediastinum. Following pneumonectomy, the post-surgical space gradually fills with fluid with replacement of air over time (Fig. 11). Obliteration of the post-pneumonectomy space and herniation of normal lung across the midline usually take weeks to months [28]. Post-pneumonectomy syndrome is described in children and young adults after right pneumonectomy. It results from hyperinflation of the left lung with herniation across the midline towards the right side [29]. This herniation results in stretching and compression of the left mainstem bronchus with narrowing of the trachea and left bronchus between the pulmonary artery and descending thoracic aorta (Fig. 12a). Tissue expanders or silicone breast implants are used in the post-pneumonectomy space to prevent such transmediastinal herniation. Additional or larger implants may be needed over time in growing children to treat this entity (Fig. 12b).

### Transdiaphragmatic hernia

The diaphragm is a dome-shaped structure with a central tendon and circumferential muscular fibres arranged in three groups: pars lumbaris, pars costalis and pars sternalis. Gaps between the muscular layers are only covered by pleura, peritoneum and fascial layers, resulting in potential sites of weakness [30]. Diaphragmatic hernias can be either mediastinal or intrapleural [31]. Mediastinal trans-diaphragmatic hernias can be in the prevascular space (Morgagni and Larry’s space) or visceral compartment (pericardial hernia, hiatal hernia) [35]. Bochdalek hernia is an example of an intrapleural hernia. Post-traumatic hernias may not follow these strict boundaries as they can result from tears in both the mediastinal and pleural portions of the diaphragm.

#### Morgagni hernia

**Fig. 13:**
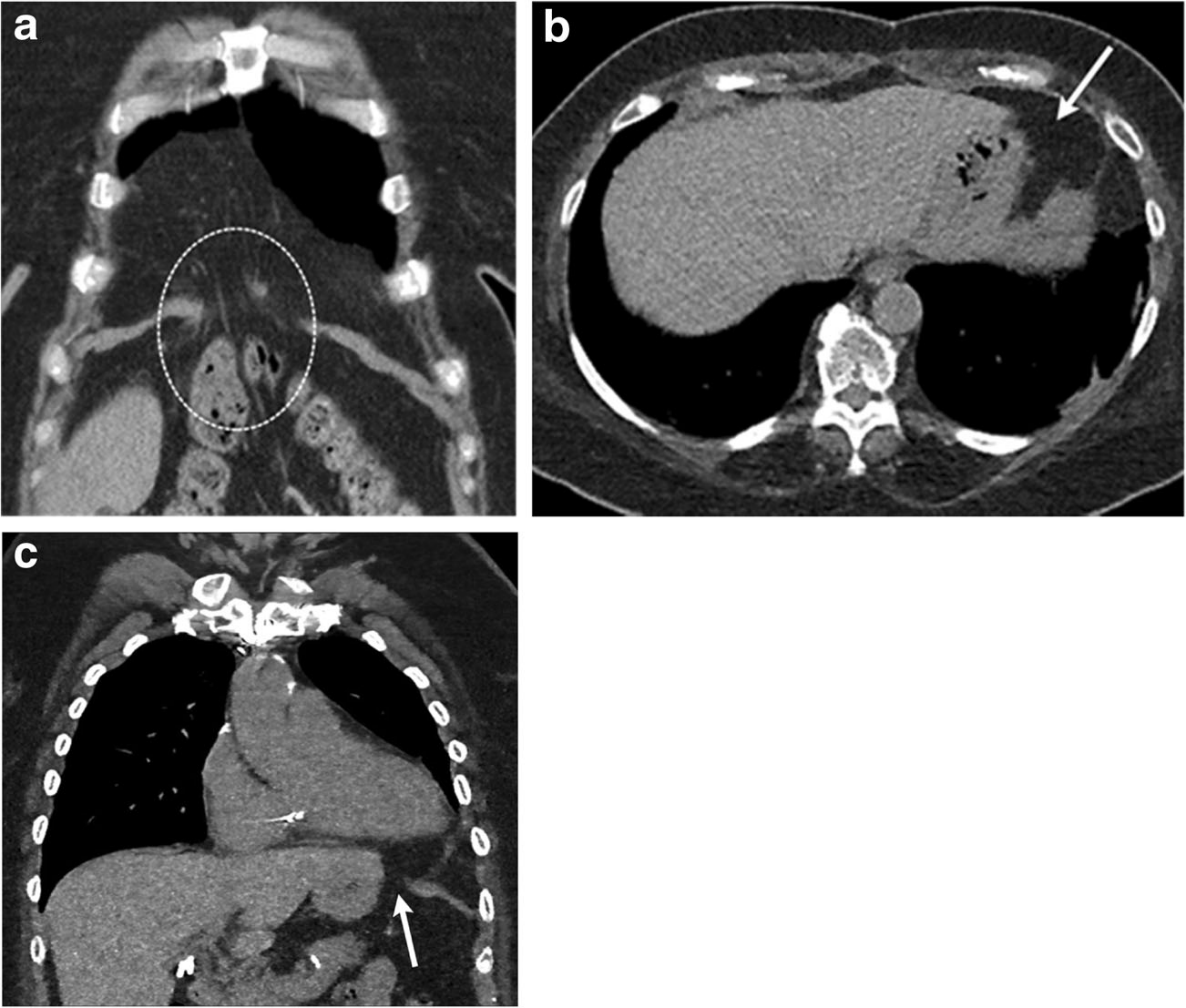
**a** A 46-year-old female being evaluated for right cardiophrenic angle opacity identified on a chest radiograph obtained during evaluation for pneumonia. Coronal CECT of the thorax identifies herniation of the omentum (dotted circle) through a clear defect in the right anterior-medial diaphragm (**a**) consistent with a Morgagni hernia. **b**, **c** A 49-year-old patient in the emergency department after trauma. Supine chest radiographs were suspicious for widening of the mediastinum. Axial (**b**) and coronal (**c**) CT clearly demonstrates a focal defect in the left anterior-medial diaphragm (white arrow) with herniation of peritoneal fat into the pericardium

A Morgagni hernia is characterised by a small anatomical defect in the space between the pars costalis and pars sternalis on the right side of the diaphragm. This potential space, also known as the sternocostal triangle, is bordered by the sternum, diaphragm and pericardium and contains internal thoracic vessels and lymphatics (Fig. 13a). The incidence of congenital Morgagni hernias is < 3% of live births [32] and 12% of diaphragmatic defects identified in infancy [33]. A similar gap on the left side of the diaphragm is Larry’s space (Fig. 13b, c). Regardless of laterality, these are called Morgagni hernias.

On CXR, a Morgagni hernia presents as an opacity in the cardiophrenic angle. Differential diagnosis includes a prominent fat pad, lymphadenopathy, and bronchogenic or pericardial cyst. On CT, the defect in the sternocostal triangle is usually identified containing the omentum in adults, but it may contain the liver, bowel loops or stomach in children. MRI is used in challenging cases and distinguishes herniated contents as liver vs. mass or metastasis.

#### Intrapericardial diaphragmatic hernias

**Fig. 14:**
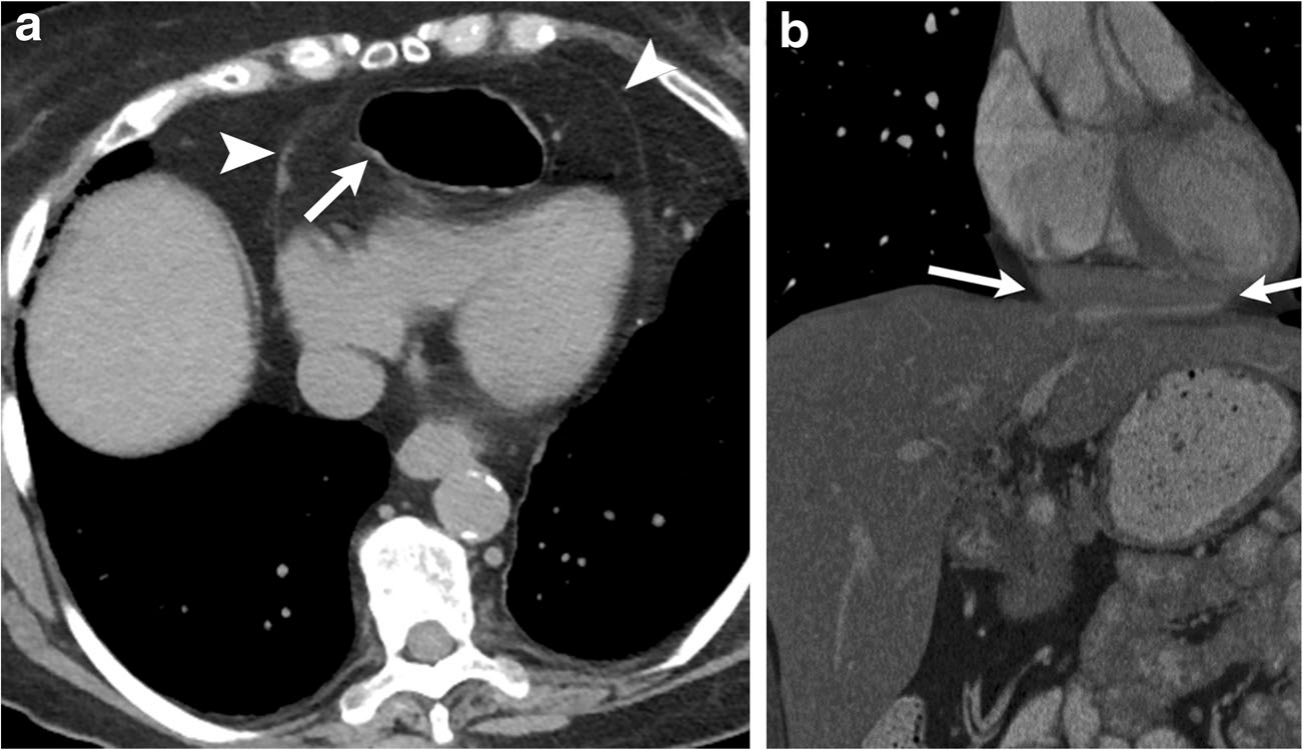
**a** A 44-year-old male with a recent history of blunt abdominal trauma, now presenting to the emergency department with upper abdominal pain. Axial CT with oral contrast identifies herniation of the stomach (white arrows) into the pericardium (arrowhead) with mild mass effect over the right ventricle. **b** A 56-year-old male with a remote history of blunt abdominal trauma and a recent diagnosis of Hodgkin's lymphoma. A pericardial mass was identified on a recent echocardiogram. Coronal CECT identifies herniation of the left lobe of the liver in the pericardium (white arrows). There is minimal mass effect over the right ventricle

Intrapericardial diaphragmatic hernias are rare and mostly the sequelae of indirect blunt trauma [34]. Abdominal contents can herniate into the pericardium through such tears. CXR can identify retrosternal air or bowel loops. CT can confirm the herniated stomach or other bowel loops (Fig. 14a). Liver can herniate into the pericardium and mimic a pericardial mass on echocardiography. CT with contrast can accurately identify the herniated viscera such as liver (Fig. 14b). MRI has high spatial and temporal resolution and can aid not only in characterising such a pericardial mass but also in evaluating for any associated pericardial constraint.

#### Hiatal hernia

**Fig. 15:**
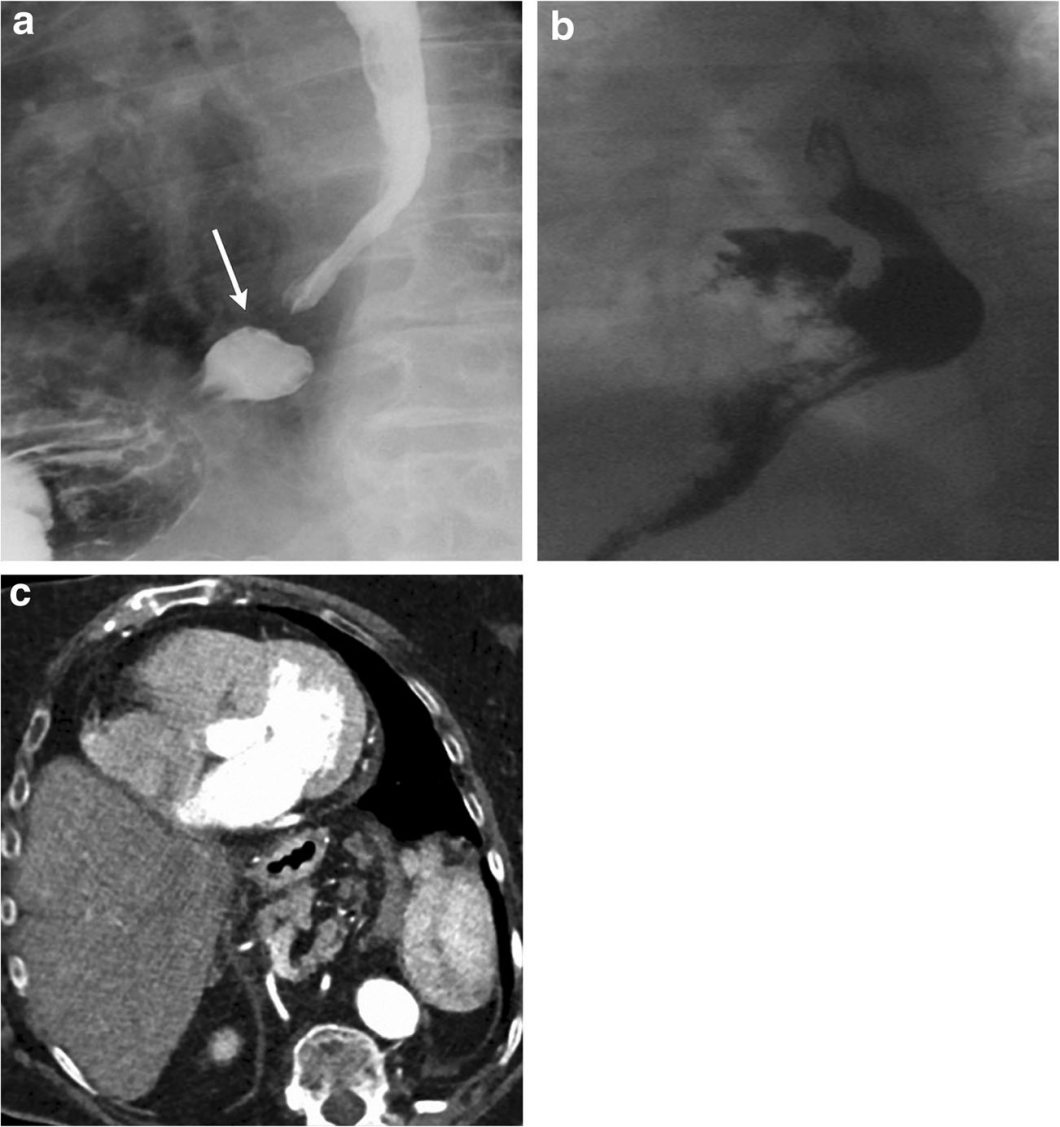
**a** A 79-year-old female with history of (h/o) heartburn on lying down after dinner. Left anterior oblique projection from the contrast oesophagogram identifies herniation of the gastro-oesophageal junction (white arrow) into the thorax; finding is consistent with type I or sliding hiatal hernia. **b** A 72-year-old male with morbid obesity and recurrent reflux pneumonitis. Lateral projection of a barium oesophagogram identifies intrathoracic herniation of the gastro-oesophageal junction with the gastric fundus, consistent with type III or mixed para-oesophageal hiatal hernia. **c** A 78-year-old female undergoing preoperative evaluation for transcatheter aortic valve replacement. Axial contrast CT identifies a large hiatal hernia containing stomach, large bowel, splenic vessels and pancreas in the hernia sac, compatible with a type IV hiatal hernia (Movie 2)

Hiatal hernia (HH) represents herniation of the stomach into the thorax through a defect in the oesophageal hiatus [35]. Hiatal hernias are the most common diaphragmatic hernias in adults. It is estimated that > 50% of adults in the western population > 50 years of age have these hernias [36, ]. There are four types of hiatal hernias; these can be identified on UGI, CT, and MRI. Type I or sliding hiatal hernia represents the most common type in which there is intrathoracic migration of the gastro-oesophageal junction due to weakness of the phrenico-oesophageal membrane (Fig. 15a). In type II HH, the gastro-oesophageal junction remains below the diaphragm, while the gastric fundus herniates into the thorax from a focal defect in the phrenico-oesophageal membrane. Type III HHs are compound hernias in which the phrenico-oesophageal membrane is not only weakened and stretched, but there is also a defect in the anterolateral portion of this membrane. This results in herniation of the gastro-oesophageal junction and gastric fundus into the thorax (Fig. 15b). These are the most common form of para-oesophageal hernias and can be associated with gastric rotation. Type IV hernia is characterised by type III hernia along with herniation of other abdominal organs, which can include the pancreas, spleen and liver (Fig. 15c).

#### Bochdalek hernia

Bochdalek hernia occurs through defects in the pars lumbaris and pars costalis and is more common on the left side. These are the most common congenital diaphragmatic hernias, with an estimated incidence of 1 per 2000–5000 live births [33]. In adults, Bochdalek hernias are often underreported and can be identified in 0.17% [37] to 6% [38] of patients. Acquired hernias represent defects or tears in the diaphragm. Diaphragmatic hernias with larger defects have a higher likelihood of being acquired after blunt trauma or a vigorous bout of coughing in a paralysed or thin hemidiaphragm rather than being congenital defects in the diaphragm.

**Fig. 16:**
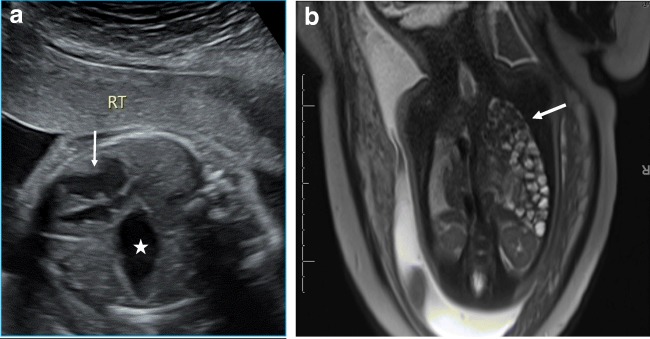
**a** Axial ultrasound image of a 21-week-old foetus identifies stomach (star) at the level of the heart (white arrow) in the thorax. This finding is suggestive of a congenital diaphragmatic hernia. **b** Coronal images as seen on prenatal MRI (**b**) demonstrates multiple loops of fluid-filled bowel (white arrow) extending to the left thoracic apex, compatible with a Bochdalek hernia. There is associated ipsilateral lung hypoplasia

These hernias can be identified on routine prenatal US (Fig. 16a) when stomach or bowel loops are seen in the thorax. Prenatal MRI is useful in confirming the defects and assessing for lung maturity (Fig. 16b). They can be associated with ipsilateral lung hypoplasia. Commonly the abdominal contents extend across the diaphragm into the thorax because of higher intra-abdominal pressure. Larger defects in the diaphragm can lead to herniation of the bowel loops and omentum into the mediastinum.

#### Post-traumatic diaphragmatic hernia

Diaphragmatic injury due to blunt trauma can lead to complex tears that can be in the central tendon and/or muscular fibres. This can lead to herniation of abdominal contents into the thorax; this often remains clinically occult but is incidentally identified on CT or MRI. The signs on CT to assess diaphragmatic injury can be direct or indirect.

Directs signs are:

1. Segmental diaphragmatic defect: when there is a focal loss of continuity in the diaphragm.
2. Dangling diaphragm sign: inward curling of the free edge of the torn diaphragm. This forms a soft tissue attenuation curvilinear structure.
3. Absent diaphragm sign: absence of the hemidiaphragm in a region where the diaphragm is expected to be clearly identifiable.

Indirect signs are:

1. Herniation of abdominal organs or peritoneal fat into the pleural or pericardial space.
2. Collar sign: waist-like constriction of the herniated structure at the site of the diaphragmatic defect. A variation of the collar sign is the hump sign, which refers to the shape of the herniated liver located above the level of the diaphragm. On axial CT images, the band sign can be seen, which corresponds to a linear hypoattenuation that transects the herniated liver between the edges of the diaphragm.
3. Dependent viscera sign: this represents direct contact between the herniated abdominal organs and the chest wall, without any interposition of the lungs.
4. Elevated abdominal organs sign: this is produced by the displacement of abdominal structures above the level of the hemidiaphragm. Nchimi et al. [13, 23] proposed using a right hemidiaphragmatic elevation > 5 cm above the level of the left hemidiaphragm as a threshold for right-sided and a left hemidiaphragmatic elevation > 4 cm above the level of the right hemidiaphragm as a threshold for left-sided diaphragmatic rupture.

**Fig. 17:**
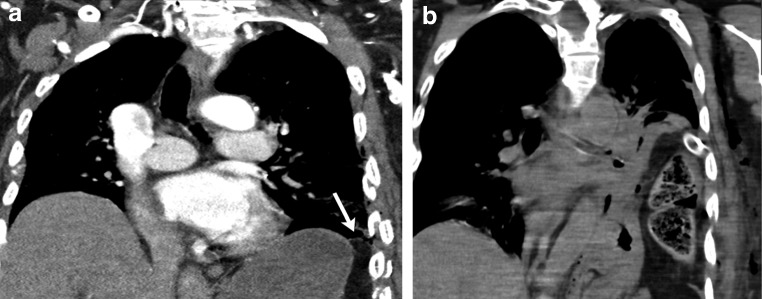
**a, b** An 80-year-old male with blunt thoracic and abdominal trauma following a motor vehicle accident. Coronal contrast CT (**b**) identifies multiple left rib fractures. In addition, there is a focal defect at the lateral aspect of the left hemidiaphragm (white arrow). The patient presented 2 days later with increasing shortness of breath and a new opacity on the frontal radiograph. Coronal image from follow-up CT (**b**) identifies interval enlargement of the left lateral diaphragmatic defect with new herniation of the stomach, splenic flexure and omentum into the left thorax. Emergent surgical repair was performed for this hernia

Occasionally there can be a delayed presentation of a diaphragmatic hernia in which a small initial tear progressively enlarges over time with subsequent herniation of the intra-abdominal contents into the thoracic cavity (Fig. 17a, b) [39].

### Thoracic extension of abdomen wall hernia

**Fig. 18:**
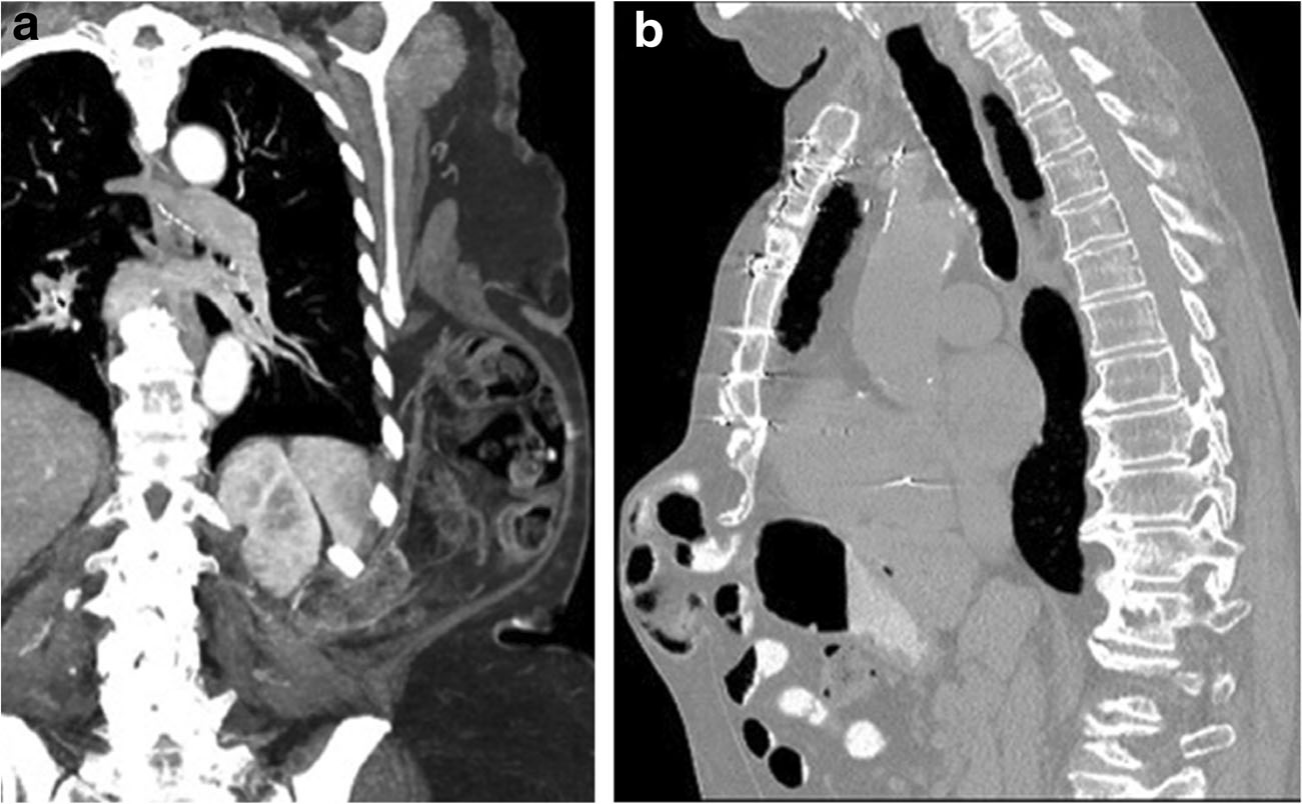
**a** An 80-year-old female being evaluated for transcatheter aortic valve replacement for severe aortic stenosis. Coronal maximum intensity projection image from a contrast-enhanced CT demonstrates a large left lateral abdominal wall hernia that originates at the lumbar triangle. The hernia sac is wide and contains small bowel, large bowel and omentum. It extends into the subcutaneous fascia of the thorax up to the level of the left 6th rib. **b** A 77-year-old male with lung cancer treated with stereotactic radiation therapy. Sagittal CECT obtained for surveillance demonstrates a large anterior abdominal wall hernia with subcutaneous extension into the pre-xiphoid portion of the anterior thorax

Abdominal wall hernias can extend above the level of the diaphragm through the superficial or deep layers of the thorax and present as a superficial chest wall hernia. These hernias can originate from either the ventral or the lateral abdominal wall. Lumbar hernias occur through defects between the 12th rib and iliac crest and are usually sequelae of prior trauma or surgery (Fig. 18a). Incisional hernias are seen in the midline anterior abdominal wall, typically as a late complication of abdominal surgery (Fig. 18b).

### Complications of thoracic hernias

Complications of thoracic hernias include trauma, obstruction, incarceration and strangulation. The herniated contents in a chest wall hernia are also at increased risk of injury from minor trauma. Obstruction can be seen in herniated bowel loops or stomach. Incarcerated hernia is characterised by an irreducible hernia because of a narrow neck and indicates that the contents of the hernia sac are irreducible [40, 41].

Incarceration predisposes to strangulation and obstruction in cases with bowel herniation. Initially there is angulation and distortion of the lymphatics followed by veins and arteries at the level of the neck of the hernia, which can cause lymphatic and venous obstruction. On CT, the herniated viscera can appear enlarged, oedematous, hypodense and with decreased contrast enhancement. If left untreated, this can lead to complete arterial occlusion and strangulation. A strangulated hernia with arterial occlusion needs to be treated emergently; otherwise it will lead to ischaemia and necrosis [41].

The complications of thoracic hernias can be best described in terms of the hernia contents.

#### Lung

**Fig. 19:**
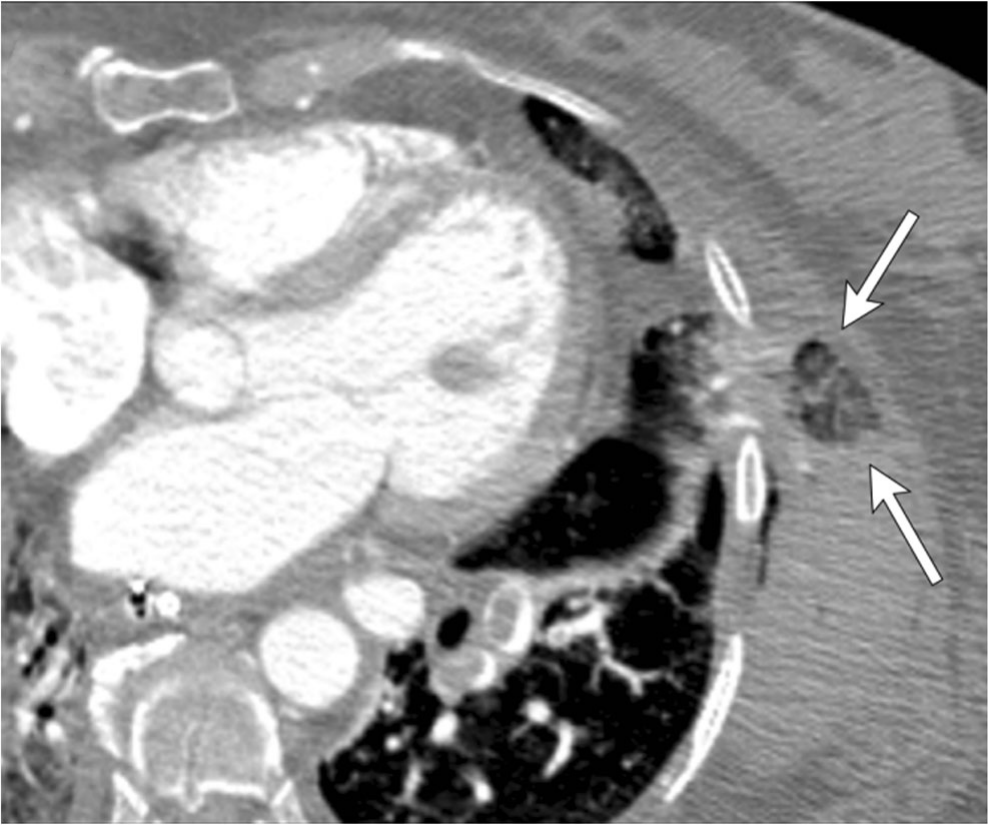
A 48-year-old patient with new onset left chest wall pain after recent lung transplantation. Left intercostal strangulated lung hernia. Axial CECT image in a patient with left lung transplant. The focal herniated left lower lateral portion of the lung is strangulated (arrows) resulting in its heterogeneous opacification. The ground-glass opacities in the lingual and sub-segmental atelectasis in the left lower lobe anterior segment can also be secondary to the left lower lobe pulmonary embolus

Incarcerated lung hernia presents as a non-reducible, well-circumscribed bulge. On CT, change in the calibre of the airways (Fig. 4a) or abrupt narrowing of the pulmonary vessels indicates impending strangulation of the herniated lung. Strangulation of lung parenchyma is rare but has been described in case reports [42]. Abrupt narrowing of the bronchi or change in the calibre of the pulmonary vessels at the neck of the hernia may be signs suggestive of impending strangulation on CT. Thrombus may be present in pulmonary arterial branches (Fig. 19) leading to a strangulated lung segment.

#### Stomach

Complications of a herniated stomach include incarceration, strangulation and volvulus. Gastric volvulus can be either organoaxial or mesentroaxial. In organoaxial volvulus, the rotation occurs along the long axis of the stomach, whereas in mesentroaxial volvulus the rotation is perpendicular to the long axis of the stomach with the displaced antrum being superior to the GE junction. Gastric rotation or a twist > 180° can lead to strangulation and obstruction [43]. Organoaxial volvulus is more common in hiatal hernia and has a high incidence of strangulation and necrosis [44]. Organoaxial positioning of the stomach refers to partial rotation < 180° without gastric obstruction.

**Fig. 20:**
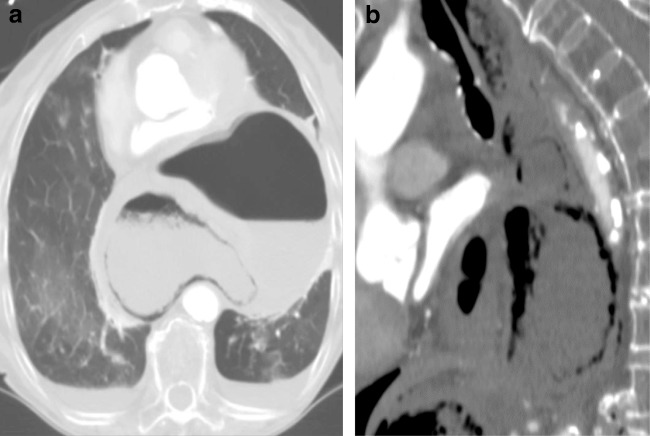
**a, b** A 92-year-old female with acute onset of severe upper abdominal pain, vomiting and nausea. Sagittal (**a**) and axial (**b**) contrast CT of the thorax (double rule out) demonstrated an intrathoracic stomach with organoaxial volvulus and gastric pneumatosis (Movie 3) requiring emergent surgical repair

On CXR, two retrocardiac air fluid levels can be seen. On UGI, findings diagnostic of an organoaxial volvulus include an intrathoracic stomach with an inferior position of the GE junction and inferiorly directed pylorus with a reverse position of the greater and lesser curvatures. In addition, inability to pass the orogastric tube or failure in the passage of oral contrast beyond the stomach indicates rotation > 180° and gastric obstruction. Often CT is obtained before UGI and can identify the herniated intrathoracic stomach and pneumatosis and locate the transition point of the gastric obstruction. Contrast-enhanced CT can also identify decreased enhancement in the gastric wall due to hypoperfusion and strangulation (Fig. 20a, b).

#### Bowel

**Fig. 21:**
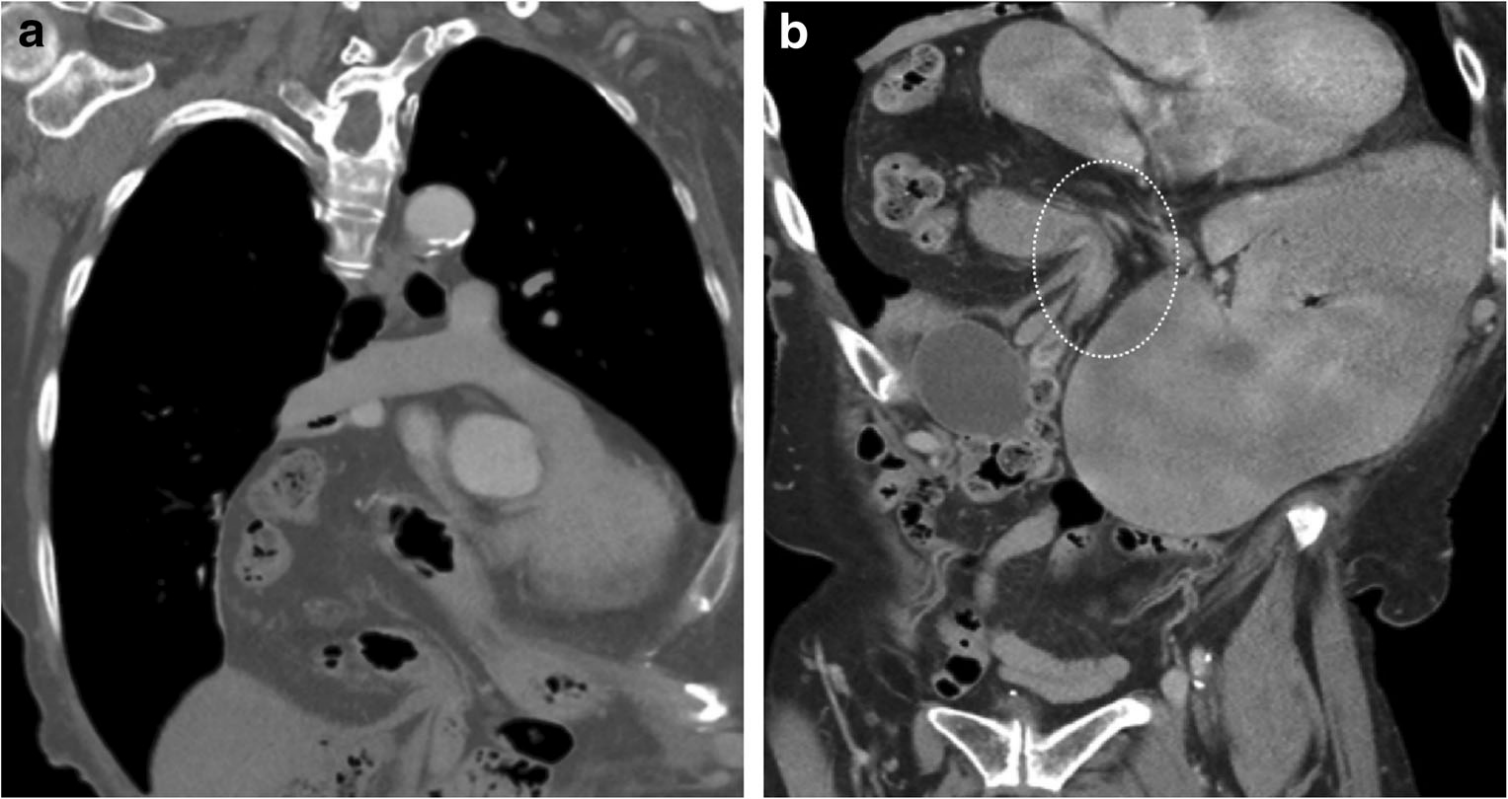
**a, b** An 86-year-old male with history of (h/o) melanoma, now presenting with chronic right upper abdominal pain. Coronal contrast CT (**a**) identifies a right retrosternal diaphragmatic hernia. Note the narrow neck of the hernia sac with herniation of the stomach (body, pylorus and antrum), colon and omentum into the mediastinum. This is an incarcerated hernia. The patient was offered elective repair, which was declined. The same patient presented with new onset of acute right upper quadrant pain after 2 months. Coronal contrast-enhanced CT (**b**) demonstrates severe dilation of the stomach with gastric outlet obstruction (Movie 4). The herniated hepatic flexure is not dilated. Adhesions within the hernia sac can lead to bowel obstruction and narrowing. These require urgent decompression of the distended stomach by placement of a gastric tube followed by surgical repair

The herniated bowel can undergo a closed loop obstruction. In such cases, CT can identify bowel wall thickening, abnormal wall enhancement, engorgement of vessels and pneumatosis. Presence of free fluid, bowel wall thickening and distention represents impeding strangulation. In patients with chronic hernia, adhesions can form in the hernia sac with fibrosis. This can lead to slowly progressive obstruction of the herniated stomach or bowel loops, which can present as acute-on-chronic obstruction (Fig. 21a, b).

#### Abdomen viscera

**Fig. 22:**
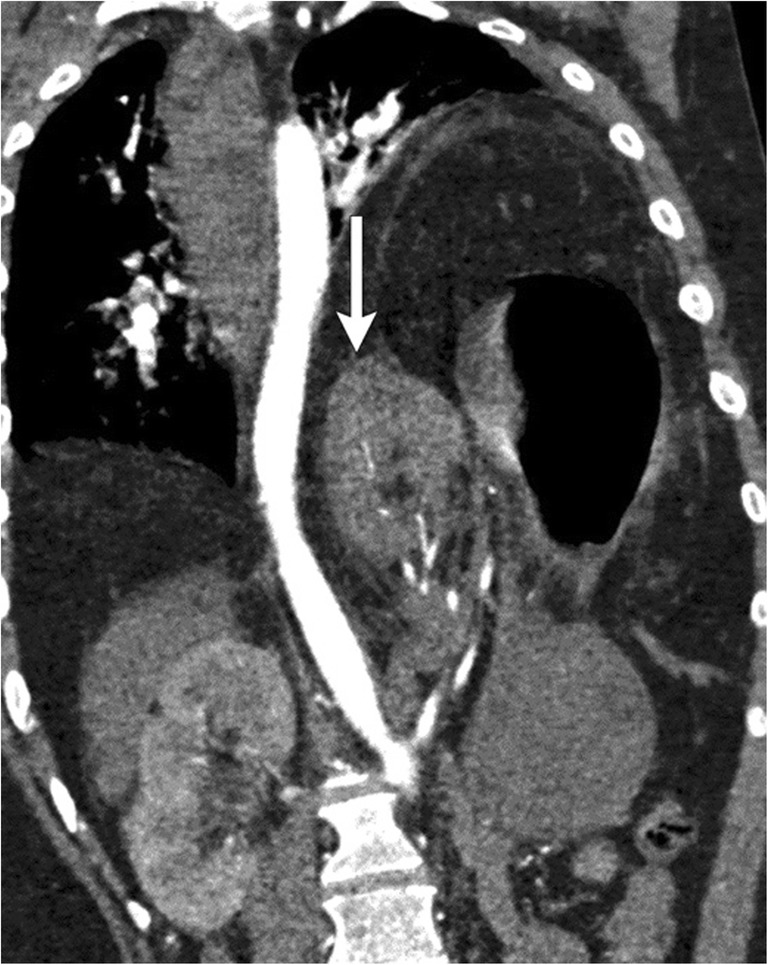
A 31-year-old patient who was on bed rest for lower extremity fracture, now presenting with new-onset chest pain following a severe bout of coughing. Coronal contrast CT demonstrates a large left diaphragmatic hernia, with left kidney, stomach and omentum present within the left thorax. Note decreased enhancement of the left kidney (white arrow) compared with the right kidney on these arterial phase images. The renal artery is normal; the renal vein cannot be evaluated on these arterial-phase images. Intraoperative findings were consistent with these findings demonstrating narrowing of the left renal vein with venous engorgement at the level of the diaphragmatic defect (Movie 5)

**Fig. 23:**
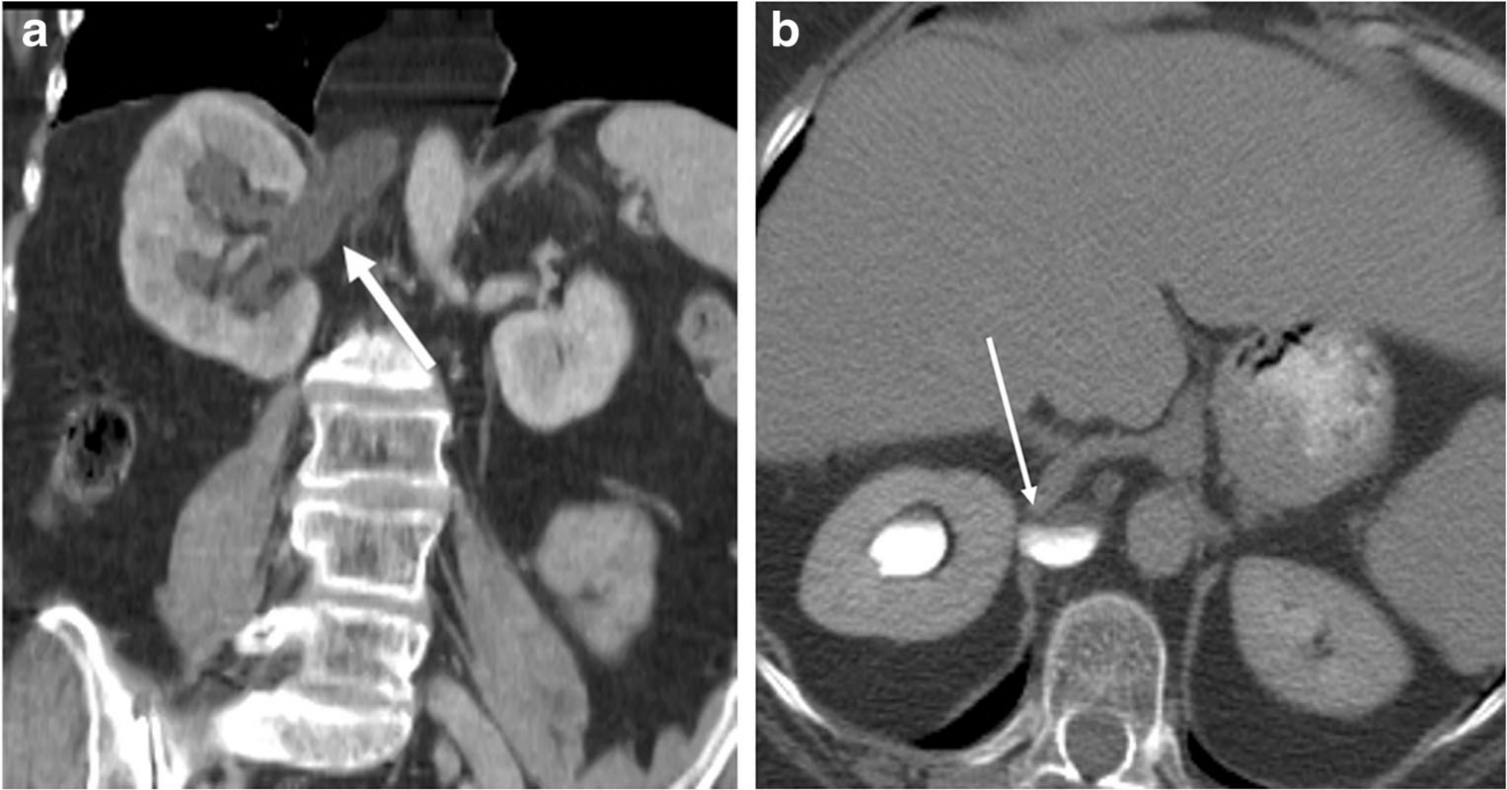
**a, b** Coronal (**a**) venous phase and axial delayed excretory phase (**b**) CT from a patient with hydroneprosis identifies a focal defect in the right posterior diaphragm (white arrow) with herniation of right ureter into the thorax. There is ureteric obstruction at the neck of the hernia with resultant hydronephrosis

Herniation of lung, kidney, spleen, bowel, etc., through small defects can lead to obstruction, vascular compromise, strangulation and ischaemia. Acute herniation through a narrow neck increases the risk for vascular compression and strangulation of herniated abdominal organs and bowel. In cases of visceral herniation of solid organs such as kidney or spleen, venous compression and obstruction may occur before arterial obstruction (Fig. 22). There can be hydronephrosis from herniation of the ureter through a congenital or acquired diaphragmatic defect (Fig. 23a, b).

## Conclusion

Thoracic hernias can occur at the thoracic inlet, chest wall or diaphragm. Imaging, particularly CT, plays an important role in establishing the diagnosis, characterising the type, delineating the extent, identifying the content, detecting complications and providing a roadmap for interventions.

## Electronic supplementary material

Movie 1 Lung hernia. Axial CT in a patient with previous right upper lobe wedge resection for a stage I lung cancer identifies focal intercostal herniation of the right upper lobe. (MP4 4032 kb)

Movie 2 Hiatal hernia. Axial contrast-enhanced CT identifies a large hiatal hernia containing stomach, large bowel, splenic vessels and pancreas in the hernia sac. This is a type IV hiatal hernia. (MP4 5772 kb)

Movie 3 Gastric volvulus. Contrast-enhanced CT of the thorax demonstrates intrathoracic stomach with organoaxial volvulus and gastric pneumatosis. (MP4 10600 kb)

Movie 4 Gastric outlet obstruction. Contrast-enhanced CT demonstrates herniation of the stomach into a Morgagni hernia with severe dilation of the stomach with gastric outlet obstruction (MP4 4021 kb)

Movie 5 Traumatic diaphragmatic hernia. Contrast-enhanced CT demonstrates a large left diaphragmatic hernia with left kidney, stomach and omentum present within the left thorax. Note the decreased enhancement of the left kidney. (AVI 5472 kb)
